# Gallium Nitride for Space Photovoltaics: Properties, Synthesis Methods, Device Architectures and Emerging Market Perspectives

**DOI:** 10.3390/mi16121421

**Published:** 2025-12-18

**Authors:** Anna Drabczyk, Paweł Uss, Katarzyna Bucka, Wojciech Bulowski, Patryk Kasza, Paula Mazur, Edyta Boguta, Marta Mazur, Grzegorz Putynkowski, Robert P. Socha

**Affiliations:** 1 CBRTP SA Research and Development Center of Technology for Industry, Zygmunta Modzelewskiego 77 St., 02-679 Warszawa, Poland; 2 Faculty of Non-Ferrous Metals, AGH University of Krakow, Mickiewicza 30 Av., 30-059 Kraków, Poland

**Keywords:** Gallium nitride (GaN), wide bandgap semiconductors, space photovoltaics, radiation hardness, InGaN/GaN heterostructures, epitaxial growth, multijunction solar cells

## Abstract

Gallium nitride (GaN) has emerged as one of the most promising wide-bandgap semiconductors for next-generation space photovoltaics. In contrast to conventional III–V compounds such as GaAs and InP, which are highly efficient under terrestrial conditions but suffer from radiation-induced degradation and thermal instability, GaN offers an exceptional combination of intrinsic material properties ideally suited for harsh orbital environments. Its wide bandgap, high thermal conductivity, and strong chemical stability contribute to superior resistance against high-energy protons, electrons, and atomic oxygen, while minimizing thermal fatigue under repeated cycling between extreme temperatures. Recent progress in epitaxial growth—spanning metal–organic chemical vapor deposition, molecular beam epitaxy, hydride vapor phase epitaxy, and atomic layer deposition—has enabled unprecedented control over film quality, defect densities, and heterointerface sharpness. At the device level, InGaN/GaN heterostructures, multiple quantum wells, and tandem architectures demonstrate outstanding potential for spectrum-tailored solar energy conversion, with modeling studies predicting efficiencies exceeding 40% under AM0 illumination. In this review article, the current state of knowledge on GaN materials and device architectures for space photovoltaics has been summarized, with emphasis placed on recent progress and persisting challenges. Particular focus has been given to defect management, doping strategies, and bandgap engineering approaches, which define the roadmap toward scalable and radiation-hardened GaN-based solar cells. With sustained interdisciplinary advances, GaN is anticipated to complement or even supersede traditional III–V photovoltaics in space, enabling lighter, more durable, and radiation-hard power systems for long-duration missions beyond Earth’s magnetosphere.

## 1. Introduction

With the rapid expansion of satellite constellations, lunar missions, Mars exploration and development of orbital infrastructure, the demand for lightweight, efficient, and durable power generation systems has become increasingly critical. Among the available technologies, photovoltaic (PV) devices converting solar radiation into electricity remain the primary solution for space-based power supply, owing to their modularity, long operational lifetimes, and continuous availability of solar irradiance in orbit [1,2].

However, unlike terrestrial environments, space imposes extreme and multifactorial challenges on photovoltaic materials. These include:

- Intense ionizing radiation from high-energy protons, electrons, and heavy ions, particularly in low Earth orbit (LEO), medium Earth orbit (MEO), and geostationary Earth orbit (GEO);
- Elevated ultraviolet (UV) and X-ray fluxes;
- Wide thermal cycles, with temperature variations ranging from approximately −170 °C to +120 °C within a single orbit;
- Vacuum-induced degradation and outgassing, especially under the influence of atomic oxygen (AO);
- Mechanical stress arising from launch vibrations and orbital thermal fatigue;
- Long mission durations requiring PV systems to operate reliably for 10–20 years without maintenance.

In addition to environmental robustness, next-generation PV materials must satisfy strict mass, volume, and efficiency constraints, particularly for interplanetary probes, small satellites, and large-scale satellite constellations. Achieving high power-to-weight ratios and compact architectures is essential for reducing launch costs and increasing mission flexibility [3,4,5]. Furthermore, with rising interest in megaconstellations and space-based solar power, scalability and cost-effective manufacturability of PV materials have become central concerns, as thousands of deployable solar units will be required to meet future energy and communication demands [6].

Over the past decades, III–V compound semiconductors including gallium arsenide (GaAs) and indium phosphide (InP) have dominated the landscape of space photovoltaics. Their high energy conversion efficiencies as well as compatibility with multi-junction devices make them appropriate for space missions, especially when combined with lattice-matched layers and spectrum-optimized designs [7,8,9].

**Figure 1:**
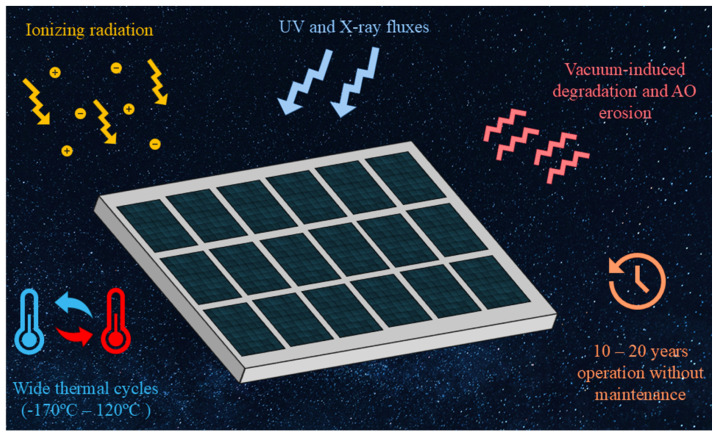
Schematic representation of critical stressors affecting III–V photovoltaic devices in space applications.

Although more radiation-tolerant than silicon, GaAs and InP still undergo significant performance degradation when exposed to prolonged bombardment by protons and electrons, which constitutes a critical challenge for long-duration missions beyond Earth’s protective magnetosphere. Moreover, their performance declines at elevated operating temperatures while their structural stability under repeated thermal cycling remains limited. The main environmental stress factors leading to the degradation of III–V photovoltaic devices in space environments are illustrated in Figure 1.

These factors collectively contribute to performance degradation and reduced structural stability of III–V semiconductors under extreme space conditions. Another serious concern is the toxicity and limited availability of key elements such as arsenic, cadmium and indium, which not only introduce environmental and safety risks but also complicate large-scale supply chains due to geopolitical and resource limitations [10,11,12]. Furthermore, the fabrication of high-quality III–V structures typically relies on epitaxial growth methods. They, in turn, require expensive vacuum equipment, rigorous process control and are often restricted to small-scale batch production. These factors collectively hinder the scalability and cost-effectiveness of these technologies, particularly in the context of mass deployment for satellite constellations and long-range space missions. These obstacles have catalyzed a growing interest in alternative PV materials capable of maintaining the performance advantages of III–V semiconductors while overcoming their inherent limitations—especially under the extreme conditions characteristic for space environments [13,14,15,16].

GaN, a wide-bandgap III-nitride semiconductor, has emerged as a highly promising material for space photovoltaics. Originally developed for light-emitting diodes (LEDs) and high-frequency electronics, GaN is now gaining attention for its exceptional combination of radiation resistance, thermal and chemical stability and interesting electronic properties. Its wide bandgap (~3.4 eV) allows for efficient operation under intense solar radiation, while its robustness against proton and electron irradiation makes it appropriate for long-duration missions in difficult and demanding orbital environments [17,18,19]. In addition to its inherent material advantages, GaN supports a range of advanced photovoltaic devices including such architectures as InGaN/GaN heterojunctions, compositionally graded structures and quantum wells [20,21].

From a technological viewpoint, recent progress in metal–organic chemical vapor deposition (MOCVD), molecular beam epitaxy (MBE), ALD, and hydride vapor phase epitaxy (HVPE) allows precise GaN film deposition on various substrates, including silicon, sapphire and native GaN—expanding the design space for integration and deployment [22].

Taken together, these features make GaN not merely a substitute for existing photovoltaic materials but a transformative and scalable platform for the next generation of solar energy harvesting systems designed specifically for the demands of space. The following sections of this review explore in detail the material properties, synthesis strategies and cell architecture that form the basis for the development of GaN-based space photovoltaics.

## 2. Key Properties of GaN Relevant to Space Applications

**Figure 2:**
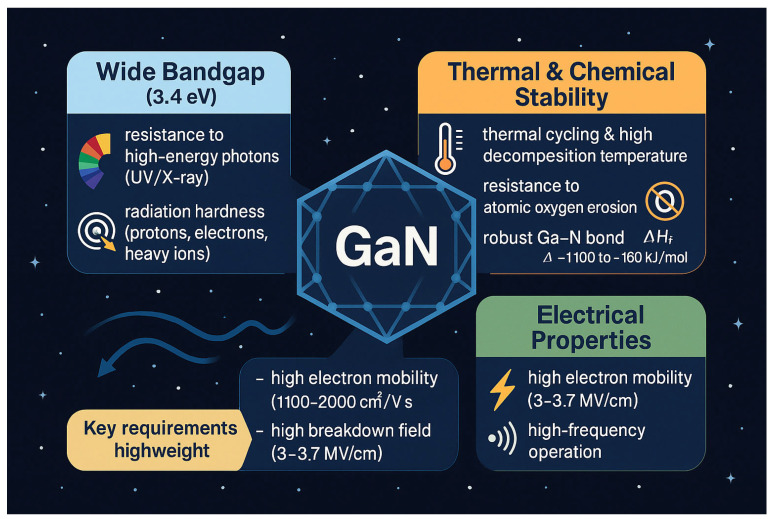
Overview of the fundamental material properties of GaN relevant to space environments.

GaN has emerged as one of the most promising semiconductor materials for next-generation space technologies. Its intrinsic properties combine a wide bandgap with remarkable thermal, chemical, and electrical stability, enabling reliable operation under extreme conditions such as intense radiation, high vacuum, and severe thermal cycling. Unlike conventional III–V semiconductors, GaN provides a unique balance of robustness and efficiency, which makes it highly relevant for both photovoltaic and electronic applications in space. The key properties of GaN relevant to space applications are summarized in Figure 2.

These attributes collectively explain the growing interest in GaN as an enabling material for space systems. They not only address the inherent limitations of traditional III–V semiconductors but also open new opportunities for designing devices with enhanced durability and performance. In the following subsections, each of these key properties is discussed in detail, with a focus on their implications for space photovoltaics and electronics.

### 2.1. Wide Bandgap and Resistance to Radiation-Induced Damage

A significant characteristic making GaN exceptionally promising for space photovoltaics is its wide direct bandgap of approximately 3.4 eV. This property plays an important role in determining thermal, electronic and optical stability of the material under harsh orbital conditions. In contrast to narrow-bandgap semiconductors such as Si (1.1 eV) or GaAs (1.4 eV), GaN maintains low intrinsic carrier concentrations and is significantly less susceptible to thermally induced carrier excitation [23,24]. As a result, GaN-based devices show reduced leakage currents and superior operation in high-temperature and high-irradiance environments such as low Earth orbit (LEO), geostationary orbit (GEO) and interplanetary space [25]. The wide bandgap also enables an exceptional resistance of GaN to radiation-induced damage. In space, semiconductors are permanently exposed to high-energy particles which can displace atoms in the crystal lattice and generate defects acting as recombination centers. GaN, however, benefits from strong covalent bonding, low atomic number and high cohesive energy, which contribute to its resistance to displacement damage. Reported displacement threshold energies typically exceed 10 eV (around 12 eV for N and over 20 eV for Ga), significantly higher than in many III–V semiconductors such as GaAs or InP [26,27].

Numerous irradiation studies have demonstrated that GaN retains its optoelectronic properties under extreme conditions. GaN-based heterostructures and quantum wells show minimal degradation in carrier mobility, photoluminescence intensity and junction integrity even after proton fluences exceeding 10^14^ cm^−2^—levels at which GaAs or InP devices experience severe performance loss [28,29]. GaN also exhibits robustness against both ionizing and non-ionizing radiation effects, as confirmed by flight-demonstrated devices in geostationary orbit [30,31]. Power-electronics-grade GaN shows high tolerance in harsh space radiation environments, outperforming Si-based MOSFETs and eliminating the need for extensive shielding [32,33]. This radiation hardness provides system-level advantages: GaN-based devices can maintain functionality with minimal shielding, reducing both mass and volume—two critical constraints in aerospace design. Enhanced durability also lowers the risk of system failure and enables more aggressive mission trajectories, including operation in radiation-intensive environments such as lunar or Martian orbits [34].

### 2.2. High Thermal and Chemical Stability

GaN demonstrates notable thermal and chemical stability, important for reliable operation in extreme space conditions. Because of its wide bandgap and strong bonding, GaN shows good tolerance to temperature fluctuations and limited chemical degradation under vacuum and elevated temperatures. In situ decomposition studies indicate that GaN begins to decompose in vacuum only at high temperatures, while nitrogen-rich environments significantly suppress this effect [35]. Surface studies of GaN(0001) reveal that Ga-terminated surfaces resist many chemical reactions, supporting overall environmental robustness [36]. Device-level tests also confirm high thermal durability: GaN HEMTs with improved heat-dissipation structures show reduced performance degradation at elevated junction temperatures [37], and studies of deep-level traps in MOCVD-grown GaN suggest that many defect states remain stable under moderate annealing [38]. GaN exhibits high thermal stability due to its wide bandgap and strong covalent bonding in the wurtzite structure. The material maintains structural integrity at temperatures far exceeding those tolerated by GaAs or InP, which often experience lattice degradation or defect activation at much lower temperatures. The high thermal conductivity of GaN, typically 130–230 W·m^−1^·K^−1^ (with record values near 300 W·m^−1^·K^−1^), further improves heat dissipation and reduces thermally induced degradation in spaceborne devices [39,40,41].

Recent studies provide quantitative insight into the relationship between temperature cycling behavior and device performance in GaN-based electronics. In particular, experimental investigations reported in [42] directly analyze the impact of repeated thermal cycling on the electrical stability of GaN devices, showing that cyclic heating and cooling can induce gradual changes in series resistance and leakage current due to thermally induced stress at heterointerfaces. These effects are closely related to thermal-expansion mismatch and stress accumulation during repeated temperature excursions, which are especially relevant for space environments characterized by frequent day–night transitions. Complementary evidence is provided by high-temperature power-cycling studies of p-GaN HEMTs reported by Hein et al. [43]. Devices were subjected to repetitive power and temperature cycling with virtual junction temperatures reaching 175 °C and endured more than 40 million cycles without irreversible degradation of key electrical parameters. Although the on-state resistance increased with temperature—reaching approximately 2.5× its room-temperature value at 200 °C—this behavior was reversible and attributed to phonon-limited transport rather than permanent structural damage. Importantly, leakage currents at 600 V remained below 100 µA at elevated temperature, and no significant drift in threshold voltage or gate characteristics was observed. Thermal runaway occurred only under current-saturation conditions, linking performance degradation to electrothermal operating regimes rather than intrinsic material failure. These results demonstrate the inherent thermal robustness of GaN-based devices and their strong tolerance to repeated temperature cycling, which is a key advantage for space applications.

GaN’s hybrid ionic–covalent bonding contributes to its strong Ga–N bonds (bond length ~1.95 Å) and relatively high formation enthalpy (−110 to −160 kJ/mol), both indicators of thermodynamic stability [44,45,46]. Its low thermal expansion coefficients—~5.6 × 10^−6^ K^−1^ along the a-axis and ~3.2 × 10^−6^ K^−1^ along the c-axis—help minimize thermal-mismatch stress during heating–cooling cycles, reducing risks of delamination or cracking in GaN-based devices [47]. Thermodynamic studies also show that GaN resists oxidation under low oxygen chemical potential, and full oxidation becomes favorable only under harsher conditions [48]. Experimental and modeling studies further indicate that GaN layers maintain structural coherence under repeated thermal cycles up to several hundred degrees Celsius, with manageable strain relative to substrates such as sapphire or SiC [49,50,51,52].

### 2.3. Electrical Transport Properties and Breakdown Field Strength

GaN is widely recognized for its excellent electrical transport properties, which make it a leading material for high-power and high-frequency applications. Its wide bandgap and strong polar bonding give rise to high electron mobility, high saturation velocity, and an exceptionally high breakdown field. Electron mobility in GaN is governed by several scattering mechanisms, including ionized impurities, polar optical phonons, piezoelectric scattering, and acoustic phonons. At low temperatures (<100 K), acoustic phonons dominate, while at higher temperatures, polar optical phonon scattering becomes the primary limitation [53,54]. Typical room-temperature mobility values for bulk GaN lie between 1100 and 2000 cm^2^/V·s [55]. Mobility decreases with temperature due to enhanced phonon scattering, consistent with Monte Carlo and Hall measurements. GaN also exhibits a high saturation electron velocity of about 2–3 × 10^7^ cm/s at room temperature, enabling fast switching performance in electronic devices [56]. A defining property of GaN is its very high breakdown electric field, substantially exceeding that of Si or GaAs. Critical field strengths for wurtzite GaN are typically 3.0–3.75 MV/cm [55], allowing GaN devices to sustain large voltage biases. Simulations show that the fields required for drift-velocity saturation or significant electron heating increase with temperature, reaching >100 kV/cm at elevated conditions. Experimental studies confirm that GaN tolerates extreme electric fields because of its wide bandgap and low intrinsic carrier concentration. Temperature strongly affects electron transport: as the lattice heats, phonon populations rise, reducing mobility and saturation velocity. This is particularly relevant for power electronics, where self-heating is significant. Simulations show stable mobility in the low-field region, saturation at moderate fields due to polar optical phonons, and intervalley scattering at very high fields (>200 kV/cm), which alters drift velocity and energy relaxation [53,56,57].

The combination of high breakdown field, high mobility, and high saturation velocity makes GaN highly suitable for power electronics, RF amplifiers, and high-frequency systems. Its ability to function under high electric fields and elevated temperatures, together with low intrinsic carrier concentration and good thermal conductivity (~1.6 W/cm·K), enhances device reliability in demanding environments [58,59]. Table 1 summarizes key properties of GaN relative to other III–V materials, highlighting its superior bandgap, thermal conductivity, radiation tolerance, and reduced toxicity—factors contributing to its growing relevance for next-generation space photovoltaics.

The data clearly underline that GaN offers a unique balance of wide bandgap, high thermal conductivity, and superior resistance to radiation damage compared to GaAs and InP. At the same time, challenges related to substrate cost and defect management remain critical barriers for its broader deployment. These considerations set the stage for the following section, which focuses on epitaxial growth techniques and defect-reduction strategies essential for advancing GaN-based space photovoltaics.

GaN is not only relevant as a photovoltaic absorber but also as a key material for high-efficiency power-conversion circuits in space systems. Its wide bandgap, high breakdown field, and high electron mobility enable the fabrication of GaN HEMTs and Schottky diodes that operate at high frequencies with low switching and conduction losses. Such devices are central to power-management units, DC–DC converters, and MPPT circuits in satellites, where thermal constraints and radiation exposure are critical challenges. Owing to its strong resistance to displacement damage and ionizing radiation, GaN allows compact, efficient, and robust power-conditioning electronics that complement GaN-based photovoltaic absorbers.

Beyond these transport parameters, the above-described material characteristics—wide bandgap, strong bonding, high thermal stability, and excellent radiation resistance—directly reinforce GaN’s electrical performance in space environments. They allow GaN devices to maintain stable mobility, low leakage, and predictable switching behavior even under high fields, strong irradiation, and large temperature swings characteristic of orbital operation. As a result, GaN circuits used in power management, signal conditioning, and high-frequency communication systems benefit from reduced degradation and increased operational lifetime compared to conventional III–V technologies. This link between intrinsic material robustness and reliable charge transport is one of the main reasons why GaN is considered a key platform not only for photovoltaic absorbers but also for the accompanying high-efficiency electronics required in modern space missions [63,64,65].

## 3. Synthesis Techniques for GaN-Based Photovoltaic Structures

For space photovoltaics, GaN growth methods should be discussed beyond basic deposition aspects. Key factors include crystalline quality, defect density, scalability, and stability under radiation and temperature cycling. Space applications require low dislocation densities, good structural uniformity, and mechanical stability during repeated thermal changes. These requirements strongly influence which growth techniques are practically suitable. Therefore, ALD, MOCVD, MBE, and HVPE are discussed below with a focus on their relevance and limitations for GaN-based space photovoltaic devices.

### 3.1. Atomic Layer Deposition (ALD)

Atomic Layer Deposition (ALD), including plasma-enhanced variants, is characterized by excellent conformality and precise thickness control at the atomic scale. However, its inherently low deposition rates make ALD unsuitable for the growth of micron-thick photovoltaic layers. In the context of space photovoltaics, ALD is therefore best positioned as a complementary technique for ultrathin passivation layers, tunneling junctions, or interface engineering, rather than as a primary method for layer growth [66].

**Figure 3:**
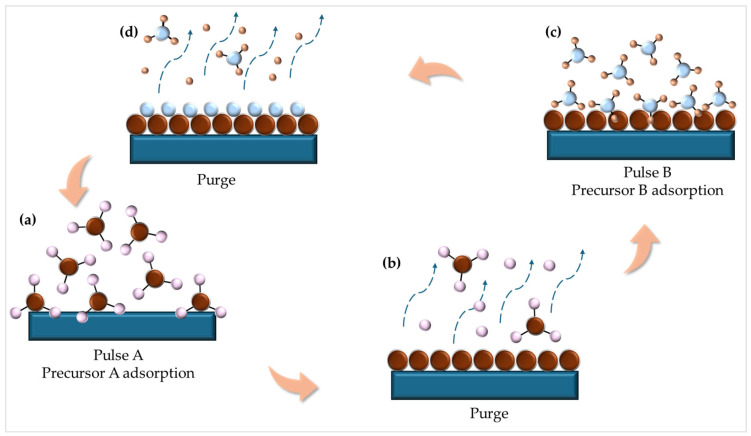
Schematic illustration of the ALD cycle. The process consists of alternating precursor pulses separated by purge steps: (**a**) precursor A adsorption on the activated substrate surface, (**b**) purge to remove unreacted molecules and by-products, (**c**) precursor B adsorption and surface reaction, followed by (**d**) a second purge. Each cycle deposits a controlled sub-monolayer, enabling atomic-scale thickness control and conformal film growth.

ALD is a vapor-phase technique based on self-limiting surface reactions, offering atomic-scale thickness control, excellent conformality, and uniform coverage over complex three-dimensional topographies [67,68,69]. These advantages make ALD particularly attractive for the deposition of GaN thin films, especially in applications requiring ultrathin, uniform layers—such as nanoscale electronics, optoelectronics, and photovoltaics for space technologies [70,71]. In Figure 3, the ALD process is schematically presented, illustrating the sequential, self-limiting surface reactions that enable layer-by-layer growth with sub-nanometer precision.

In ALD of GaN, a typical cycle consists of alternate exposure to a gallium precursor and a nitrogen source, separated by inert gas purges to prevent gas-phase reactions. This cyclic approach allows for layer-by-layer growth, yielding stoichiometric GaN films with precise thickness control. One of the challenges in ALD of GaN is the relatively low reactivity of common nitrogen precursors (e.g., NH_3_) at low temperatures. Therefore, plasma-enhanced ALD (PEALD) is often used, where nitrogen is activated via radio frequency (RF) or microwave plasma, significantly enhancing surface reactivity and film quality at temperatures as low as 200–300 °C [72].

Various gallium precursors have been investigated in the literature for GaN ALD, including:

- Trimethylgallium (TMGa): A widely used precursor; allows for good control over film composition but may result in carbon contamination due to methyl ligands [73];
- Tris(dimethylamido)gallium (III) (Ga_2_(NMe_2_)_6_: This non-halide precursor has demonstrated promise for low-temperature processes with reduced carbon incorporation [74];
- Gallium trichloride (GaCl_3_): Although corrosive and requiring higher temperatures, it offers better control over film purity in some configurations [75];

For nitrogen sources, NH_3_ plasma and N_2_/H_2_ plasma [76] are most commonly used, enabling efficient nitridation at reduced thermal budgets—critical for temperature-sensitive substrates or multilayer structures.

An illustrative example is provided by Rouf et al. [77], who demonstrated plasma-enhanced ALD of GaN using tris(dimethylamido)gallium(III) [Ga(NMe_2_)_3_] as the gallium precursor and NH_3_ plasma as the nitrogen source. Self-limiting growth was observed in the temperature range of 130–250 °C, with a growth per cycle of approximately 1.4 Å. The resulting films exhibited crystallinity on Si(100) substrates with nearly stoichiometric Ga/N ratios and very low carbon and oxygen contamination. Notably, epitaxial GaN was achieved on 4H-SiC without the need for an AlN buffer layer—a result not previously reported for ALD GaN. The films showed an optical bandgap of ~3.42 eV and unintentional n-type conductivity. Kim et al. [78] reported thermal ALD of GaN on Si(100) substrates using GaCl_3_ and NH_3_, achieving a growth per cycle of approximately 2.0 Å/cycle. However, the thermal approach required temperatures above 400 °C and presented risks of Cl incorporation. Austin et al. [71] demonstrated high-temperature ALD of GaN on silica nanosprings using TMGa and NH_3_ at 800 °C, obtaining conformal amorphous coatings that became nanocrystalline (with an average crystallite size of 11.5 ± 0.5 nm) upon introducing Al_2_O_3_ or AlN buffer layers. Hafdi et al. [79] examined the surface chemistry of GaN ALD using TEG and NH_3_, showing via in situ mass spectrometry that ethyl ligands are removed during the TEG pulse, followed by ligand-exchange reactions during NH_3_ exposure. Banerjee et al. [70] reported a purely thermal ALD route for polycrystalline GaN using TMGa and NH_3_ at 400 °C, achieving a growth per cycle of ~0.1 nm without plasma activation and maintaining excellent conformality in high-aspect-ratio structures. The ALD window was narrow (375–425 °C), with decomposition of TMGa above 425 °C leading to contamination. Growth behavior strongly depended on NH_3_ partial pressure and pulse duration. Recent studies have expanded ALD GaN methodology. Cong et al. [80] investigated PEALD with TMGa/NH_3_ plasma over 250–350 °C, observing a transition from amorphous to oriented films. Wang et al. [81] achieved low-resistance N-polar GaN at 300 °C.

**Figure 4:**
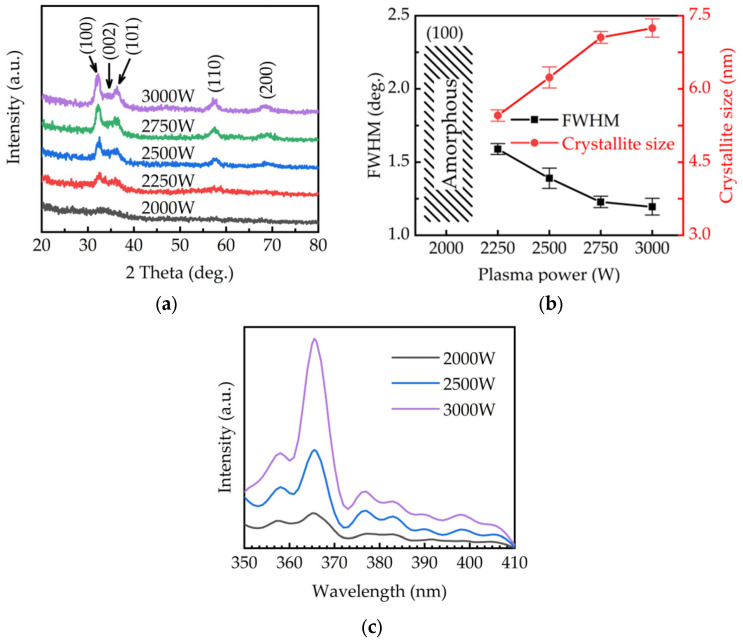
(**a**) XRD patterns of PEALD-GaN films deposited at different plasma powers, (**b**) corresponding FWHM values and crystallite sizes, and (**c**) room-temperature photoluminescence spectra showing enhanced near-band-edge emission at higher plasma power. Adapted from Ref. [82] under the CC BY 4.0 license.

Jiang et al. [82] demonstrated effective oxygen reduction in PEALD-grown GaN using NH_3_-derived plasma radicals. In Figure 4, representative XRD and photoluminescence data illustrate the direct impact of plasma power on film quality.

At low plasma power, GaN films show weak and broadened XRD peaks, indicating poor crystallinity. Increasing the plasma power leads to well-defined GaN diffraction peaks, reduced FWHM values, and increased crystallite size. Correspondingly, PL spectra reveal a strong enhancement of near-band-edge emission at ~365 nm and a reduction in defect-related luminescence, confirming improved crystalline quality and lower oxygen-related defect density. Complementary process strategies were reported by Deminskyi et al. [76], who introduced an additional B-pulse to enhance ethyl-ligand removal and increase the growth-per-cycle, and by Yun et al. [83], who demonstrated polarity control through the incorporation of an Ar-plasma buffer layer.

The reviewed studies indicate that GaN ALD/PEALD commonly relies on metal–organic precursors such as TMG, TEG, Ga(NMe_2_)_3_, and GaCl_3_, with NH_3_ as the dominant nitrogen source. PEALD enhances nitridation efficiency, reduces contamination, and enables deposition at moderate temperatures (250–350 °C), while thermal ALD typically requires ≥400 °C and may suffer impurity incorporation. Substrates include Si(100), semi-insulating GaN, 4H-SiC, and 1D architectures. Process-dependent film characteristics range from amorphous to nanocrystalline to well-oriented. Modifications such as additional reactive pulses or Ar-plasma buffers enable improved growth rates and polarity control. Overall, PEALD is emerging as the preferred approach for crystalline GaN at moderate temperatures, while thermal ALD remains advantageous for extreme conformality. Ongoing research focuses on lowering deposition temperatures, improving crystallinity without post-annealing, and minimizing impurities. Plasma diagnostics, in situ ellipsometry, and advanced characterization (XPS, TEM, XRR) are being used to better understand growth mechanisms. Emerging directions include area-selective ALD [84,85,86], hybrid ALD–CVD/MBE processes [87,88], and low-temperature ALD on flexible substrates [89], supporting future lightweight and conformable photovoltaic architectures.

### 3.2. Metal–Organic Chemical Vapor Deposition (MOCVD)

Metal–Organic Chemical Vapor Deposition (MOCVD) is the dominant and industrially established technique for the growth of GaN layers used in photovoltaic devices, particularly for the relatively thick active layers required for efficient light harvesting. Its high deposition rates (typically several µm/h), excellent thickness and composition uniformity on large-diameter wafers, and compatibility with established III–V manufacturing processes make it well suited for practical device fabrication. From the perspective of space photovoltaics, these advantages are especially important. MOCVD enables reliable growth of GaN and InGaN layers with controlled composition, sharp interfaces, and relatively low defect densities, which are key requirements for multi-junction and tandem solar-cell concepts. In addition, the method supports scalable wafer-level processing and reproducibility, both critical for space-grade devices. In contrast, techniques such as ALD, while valuable for ultrathin passivation or tunneling layers, are inherently unsuitable for micron-scale layer growth due to their very low deposition rates [90,91].

**Figure 5:**
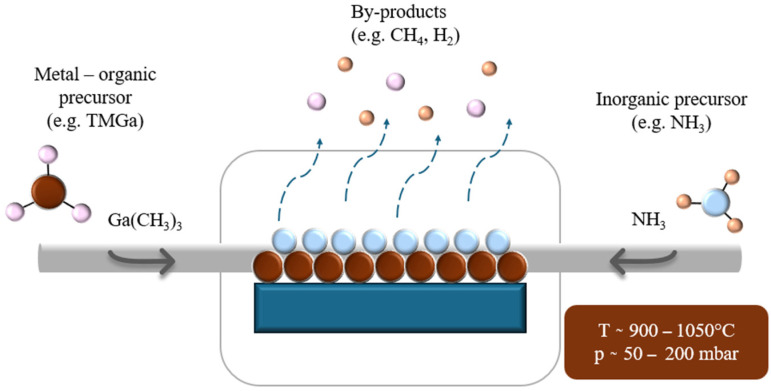
Schematic representation of the metal–organic chemical vapor deposition (MOCVD) process.

Unlike ALD, MOCVD operates under continuous gas flow rather than self-limiting surface reactions, enabling higher growth rates and the deposition of high-quality single-crystalline layers over large areas. In this process, volatile metal–organic precursors (such as TMGa and nitrogen sources (typically ammonia, NH_3_)) are introduced into a heated reactor, where they undergo thermal decomposition at the substrate surface to form the desired compound. Key process parameters—including growth temperature, V/III ratio, reactor pressure, and precursor flow rates—have a direct influence on film crystallinity, defect density, surface morphology, and polarity. In Figure 5, the principle of the MOCVD process is schematically presented, showing the continuous supply of metal–organic and nitrogen precursors and their thermal decomposition on the heated substrate surface.

MOCVD is the dominant method for GaN growth in optoelectronic and high-power electronic devices due to its scalability, ability to achieve epitaxial growth on lattice-matched and lattice-mismatched substrates, and compatibility with in situ monitoring techniques (e.g., reflectometry, mass spectrometry). Advanced variations, such as low-pressure MOCVD (LP-MOCVD), pulsed MOCVD, and selective-area growth, further expand the flexibility of this technique for both planar and nanostructured architectures.

Research on MOCVD of GaN has increasingly focused on overcoming the limitations of precursor decomposition efficiency, impurity incorporation, and surface morphology control. Various approaches have been proposed to enhance growth rates and film quality while reducing process temperatures and contamination. One such approach was presented by Zhang et al. [92], who developed and applied a laser-assisted MOCVD (LA-MOCVD) method for GaN growth. In their work, a CO_2_ laser (λ ≈ 9.219 µm) was used to resonantly excite NH_3_ molecules, significantly improving their dissociation in the reaction zone. This strategy enabled GaN growth rates of up to 10 µm/h, with smooth, pit-free surfaces even at 8.5 µm/h. The films showed a markedly reduced carbon content (~5.5 × 10^15^ cm^−3^) and high crystalline quality, with electron mobilities exceeding 1000 cm^2^/V·s at room temperature. These results demonstrated that laser-assisted NH_3_ activation can substantially enhance growth efficiency, purity, and electrical performance compared to conventional MOCVD.

Another noteworthy example is provided by Hu et al. [93], who designed and demonstrated a novel Buffered Distributed Spray MOCVD reactor intended for high-quality GaN growth for LED structures. The reactor incorporates vertically distributed gas injection and horizontal gas inlets, enabling precise and uniform precursor delivery to the reaction zone. This configuration improves gas flow control, reduces parasitic reactions, and enhances the uniformity of epitaxial layers across large wafer batches. Experimental growths performed in a 36 × 2″ wafer configuration showed that the reactor architecture supports efficient GaN and InGaN/GaN multiple quantum well (MQW) deposition with excellent thickness uniformity. Structural and optical characterization, including TEM and photoluminescence, confirmed that the BDS design yields epitaxial films of high crystalline quality, making it particularly well-suited for large-scale LED manufacturing.

Swain et al. [94] addressed the sustainability aspect of MOCVD-based GaN production by investigating the recovery of gallium from waste dust generated during the epitaxial growth process. Detailed chemical analysis of the collected reactor dust revealed a high gallium content in forms suitable for extraction. The authors developed a chemical recovery protocol involving dissolution, selective precipitation, and separation steps, ultimately obtaining gallium compounds of high purity that could be reused as precursors in subsequent growth cycles. This approach not only reduces the consumption of raw gallium but also minimizes waste generation, thereby lowering both the economic and environmental footprint of GaN manufacturing.

Delgado Carrascon et al. [95] explored a hot-wall MOCVD configuration for the homoepitaxial growth of GaN. In this reactor design, the entire chamber wall is uniformly heated rather than relying solely on localized substrate heating. This approach minimizes thermal gradients, stabilizes the reaction environment, and enhances precursor utilization efficiency. The method enabled the deposition of high-purity, defect-reduced GaN layers directly on GaN substrates, yielding atomically smooth surfaces and narrow X-ray rocking curve widths indicative of excellent crystalline quality. Such improvements are particularly advantageous for advanced optoelectronic devices, where lattice perfection and surface uniformity critically affect performance.

Collectively, these studies highlight the remarkable versatility of MOCVD as a GaN growth technique, enabling not only the fabrication of high-quality epitaxial layers for a broad range of applications but also innovations that address efficiency, scalability, and environmental sustainability. Advances in reactor design, precursor activation methods, and resource recovery strategies demonstrate that both fundamental process optimization and system-level engineering can significantly improve film quality, reduce defect densities, and lower operational costs. As such, MOCVD remains a cornerstone technology for GaN production, with ongoing research continuing to expand its capabilities for next-generation optoelectronic and power electronic devices.

### 3.3. Molecular Beam Epitaxy (MBE)

Molecular Beam Epitaxy (MBE) is a highly controlled epitaxial growth technique widely used for the fabrication of III–V compound semiconductors, including GaN. In MBE, ultra-pure elemental or compound sources are thermally evaporated or sublimated in ultra-high vacuum (UHV) conditions, producing molecular or atomic beams that impinge directly on a heated substrate. MBE offers unparalleled control over interfaces, doping profiles, and low-dimensional structures, making it highly valuable for fundamental studies and high-performance electronic devices. However, its intrinsically low growth rates and limited scalability restrict its applicability for thick photovoltaic layers required in space solar cells. As such, MBE is better suited for specialized device layers or model structures rather than large-scale photovoltaic absorbers [96].

**Figure 6:**
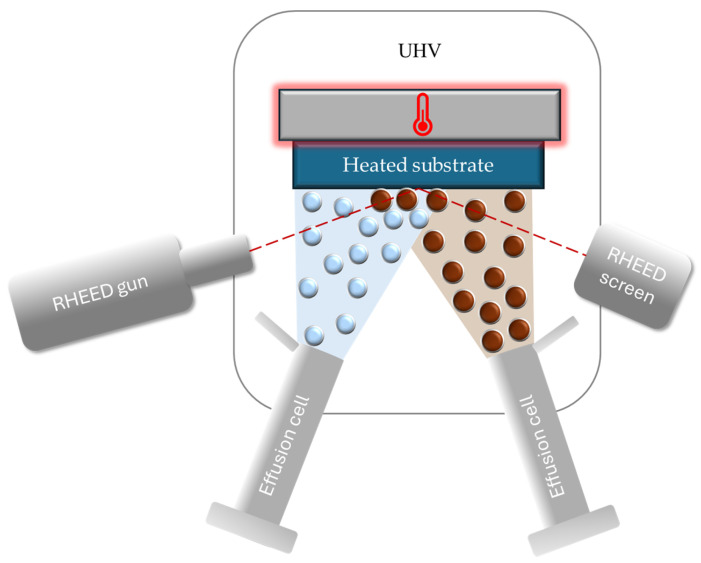
Schematic representation of the molecular beam epitaxy (MBE) process.

In Figure 6, the molecular beam epitaxy (MBE) process is schematically illustrated, highlighting the use of ultra-high vacuum, effusion cells, and molecular or atomic beams for epitaxial film growth with atomic-layer precision.

Unlike chemical vapor deposition methods, MBE relies on physical evaporation rather than chemical precursor decomposition, which allows precise control of deposition rates at the monolayer scale. The extremely low background pressure (typically below 10^−9^ Torr) minimizes impurity incorporation, enabling the synthesis of high-purity, defect-controlled films [97,98,99].

Key growth parameters—such as substrate temperature, beam flux ratios (e.g., Ga/N), and growth rate—strongly influence crystal quality, surface morphology, and defect density. The inherently slow growth rates of MBE (commonly below 2 µm/h) are offset by the unparalleled precision in controlling composition, doping profiles, and interface sharpness, making the technique particularly valuable for advanced heterostructures, quantum wells, and low-dimensional architectures. Additionally, in situ diagnostics such as Reflection High-Energy Electron Diffraction (RHEED) provide real-time feedback on surface reconstruction and growth kinetics, enabling dynamic process optimization [100,101].

A distinctive advantage of MBE lies In Its flexibility: by modifying the growth environment, energy supply, or flux modulation, researchers can directly tailor film properties, morphology, and defect density. For instance, Aggarwal et al. [102] employed laser-assisted MBE (laser-MBE) to overcome the inherent low growth rate and to stimulate enhanced reactivity of nitrogen. By optimizing the buffer layer design and pre-nitridation conditions on nonpolar r-plane (11–20) sapphire substrates, they achieved a spectrum of GaN nanostructures ranging from granular thin films (~160 nm) to vertically aligned nanocolumns (~370 nm) and nanoporous GaN films (~560 nm, pore diameters 70–110 nm). Detailed structural characterization using RHEED, high-resolution X-ray diffraction, and Raman spectroscopy confirmed the epitaxial wurtzite phase with remarkably low residual strain (0.03–0.23 Gpa). Importantly, when integrated into metal–semiconductor–metal (MSM) ultraviolet photodetectors, nanoporous GaN exhibited outstanding responsivity up to 358 mA/W at 1 V, representing a breakthrough in device performance for this morphology. This study demonstrated that MBE, when combined with laser assistance, can broaden the accessible morphological space of GaN while simultaneously enhancing optoelectronic functionality.

**Figure 7:**
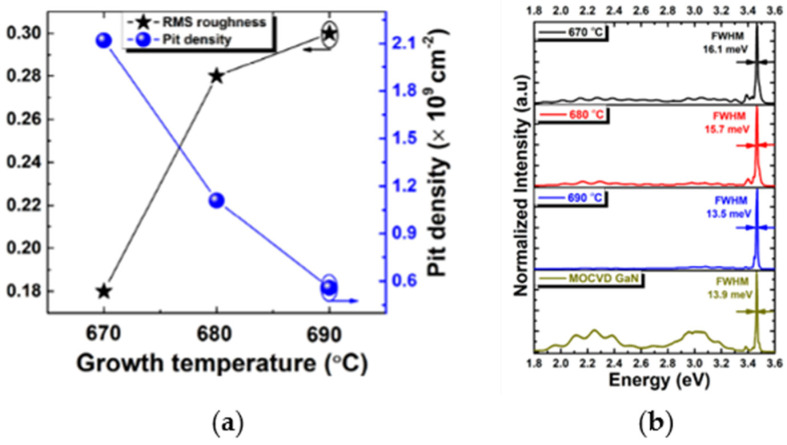
(**a**) Surface roughness and pit density of MBE-grown GaN films as a function of growth temperature, and (**b**) normalized near-band-edge photoluminescence spectra showing FWHM narrowing with increasing temperature. Adapted from Ref. [103] under the CC BY 4.0 license.

The ability of MBE to control morp”olog’cal transitions was further highlighted by Mai et al. [103], who systematically investigated the effect of substrate temperature on GaN films. Their work directly addressed the needs of HEMT structures, in which crystalline quality and defect suppression are paramount. By carefully increasing substrate temperature, they triggered a transition from three-dimensional island growth to a two-dimensional layer-by-layer growth regime. In Figure 7, the influence of growth temperature on surface morphology and defect-related optical properties is presented.

Figure 7a shows that increasing the growth temperature from 670 to 690 °C leads to a clear reduction in pit density and the formation of atomically smooth surfaces, with RMS roughness decreasing to approximately 0.3 nm at 690 °C. At the same time, Figure 7b demonstrates a progressive narrowing of the near-band-edge photoluminescence peak, with the FWHM decreasing from 16.1 meV to 13.5 meV, indicating reduced defect density and improved crystalline quality. At the optimal temperature of 690 °C, GaN films exhibited atomically smooth surfaces (RMS roughness of 0.3 nm), significant relaxation of residual strain, and strong suppression of defect-related yellow luminescence, which is commonly associated with deep-level states. Electrical characterization revealed that these high-quality MBE-grown GaN channel layers achieved breakdown voltages of ~1450 V, markedly surpassing the ~1180 V typically reported for MOCVD-grown counterparts. This result underscores the potential of MBE to engineer ultra-low-defect GaN for next-generation power electronics, where breakdown strength and reliability are critical.

Beyond temperature and morphology, alternative growth strategies within the MBE framework have been developed to further suppress defects. Yang et al. [104] introduced metal-modulated epitaxy (MME), in which the gallium flux is periodically modulated to deliberately alter surface stoichiometry and adatom kinetics. This modulation effectively regulates the competition between gallium-rich and nitrogen-rich conditions, leading to reduced incorporation of extended defects and improved uniformity across the film. By fine-tuning the modulation parameters, the authors achieved GaN layers with excellent crystalline order and reduced impurity levels. Their work demonstrated that MME can be seen as a powerful refinement of conventional MBE, capable of producing high-performance GaN films that meet the stringent requirements of optoelectronic and high-frequency devices. In turn, Suzuki et al. [105] investigated the deposition of GaN films on unconventional MgO substrates in (111) and (001) orientations using radio-frequency plasma-assisted MBE (RF-MBE). Despite the considerable lattice mismatch between GaN and MgO, the authors showed that epitaxial GaN layers with acceptable structural quality could be grown. This work highlighted the adaptability of MBE to diverse substrate platforms, expanding its potential for integration into non-standard device architectures.

In addition to morphological and structural optimization, MBE has proven highly effective for controlled doping of GaN [106]. A notable example is the introduction of magnesium acceptors during plasma-assisted MBE under shutter-controlled growth conditions, which enabled the achievement of p-type carrier concentrations as high as 3.12 × 10^18^ cm^−3^. The presence of Mg not only promoted conformal layer growth but also improved carrier mobility and overall structural quality, particularly under nitrogen-rich conditions. Such advances highlight the ability of MBE to finely tune electrical properties while maintaining excellent crystalline integrity, making it a key enabler for the development of high-performance GaN-based electronic and optoelectronic devices.

### 3.4. Hydride Vapor Phase Epitaxy (HVPE) and Ammonothermal Growth

Hydride Vapor Phase Epitaxy (HVPE) plays a crucial role in the fabrication of bulk GaN substrates due to its very high growth rates and ability to produce thick, low-defect crystals. While HVPE is generally not used for active photovoltaic layer deposition, it provides high-quality GaN substrates that significantly reduce threading dislocation densities in subsequent epitaxial layers grown by MOCVD, thereby indirectly enhancing device performance and reliability in space environments [107].

Hydride Vapor Phase Epitaxy (HVPE) and ammonothermal growth represent two of the most advanced techniques for producing bulk GaN crystals of high structural quality. HVPE, based on the chemical reaction between GaCl_3_ (generated in situ by flowing HCl over molten gallium) and NH_3_, is particularly attractive due to its exceptionally high growth rates, often exceeding 100 µm/h, and its capability to produce thick GaN layers with dislocation densities below 10^6^ cm^−2^ [108]. Optimized HVPE processes have enabled the fabrication of freestanding GaN substrates up to several millimeters in thickness, with controlled wafer curvature and reduced stress. Importantly, recent studies demonstrated that careful reactor design and gas flow engineering can yield growth uniformity variations as low as 1% across wafers up to 50 mm in diameter [109]. Moreover, laser lift-off techniques applied to thick HVPE films grown on sapphire substrates have yielded freestanding GaN with dislocation densities in the range of 2–5 × 10^6^ cm^−2^, marking a significant step toward industrial scalability [110].

In contrast, ammonothermal growth employs a solution-based approach, in which GaN is dissolved in supercritical ammonia in the presence of mineralizers (basic or acidic ammonium salts) and recrystallized onto a seed crystal under controlled thermal gradients [111]. This method is analogous to hydrothermal growth of quartz and is valued for its ability to produce crystals with extremely low dislocation densities—down to the order of 10^4^ cm^−2^—alongside narrow X-ray rocking curves (31–38 arcsec) and low optical absorption coefficients (~4 cm^−1^ at 450 nm) [112]. Detailed studies of GaN solubility and dissolution kinetics in supercritical ammonia have further clarified the role of mineralizers in enhancing mass transport and stabilizing crystal growth [108,113,114]. More recently, computational fluid dynamics (CFD) modeling has been applied to ammonothermal autoclaves, demonstrating the critical influence of convection patterns, thermal gradients, and supersaturation control on crystal size, morphology, and uniformity [115].

**Figure 8:**
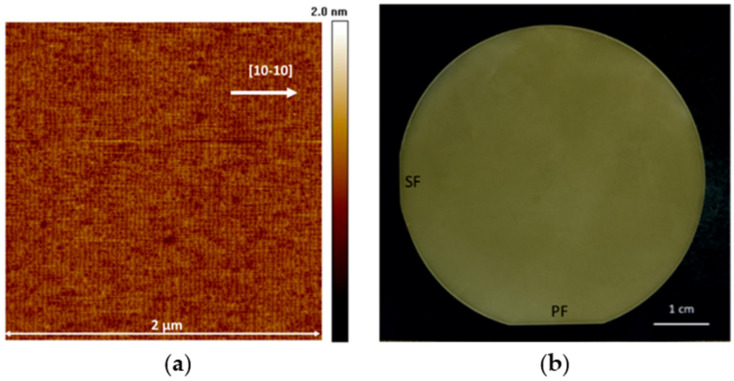
(**a**) AFM image of an as-grown ammonothermal GaN surface, demonstrating high surface uniformity and low defect density, and (**b**) photograph of a 2-inch ammonothermal GaN substrate with primary flat (PF) and secondary flat (SF) marked, illustrating wafer-scale quality and crystallographic orientation. Adapted from Ref. [111] under the CC BY 4.0 license.

Together, HVPE and ammonothermal growth offer complementary strengths: HVPE provides unmatched deposition rates and scalability for thick wafer production, while ammonothermal growth excels in defect reduction and crystalline perfection. Their continued development remains central to overcoming substrate-related challenges in GaN-based optoelectronic and power device technologies. A representative comparison of these two approaches was provided by Grabińska et al. [111], who analyzed GaN substrates obtained via HVPE and ammonothermal growth in terms of their structural, optical, and electrical properties. The authors emphasized that HVPE remains highly attractive for commercial applications, owing to its ability to deliver thick, large-diameter substrates suitable for laser diodes and power devices. At the same time, ammonothermal GaN demonstrated markedly superior structural quality, with threading dislocation densities typically in the range of 10^4^–10^5^ cm^−2^ and X-ray rocking curve widths of 31–38 arcsec, values that approach those of native GaN single crystals. In Figure 8, representative experimental images of ammonothermal GaN substrates are presented.

Figure 8a shows an AFM image of the as-grown ammonothermal GaN surface, confirming its high surface uniformity and low defect density. Figure 8b displays a photograph of a 2-inch ammonothermal GaN substrate with marked primary and secondary flats, illustrating the high macroscopic quality, wafer-scale uniformity, and technological maturity of this growth method. In addition, ammonothermal-grown crystals exhibit low optical absorption coefficients (~4 cm^−1^ at 450 nm) and high resistivity, making them particularly suitable for optoelectronic applications requiring minimal defect-related losses. Together, these results highlight the complementary nature of HVPE and ammonothermal growth: HVPE offers scalability and cost efficiency, while ammonothermal GaN provides unmatched crystalline perfection.

Building on the previously discussed bulk growth methods, Hsiang et al. [116] examined the influence of hydrogen incorporation during Molecular Beam Epitaxy (MBE) of GaN. Their study revealed that the controlled presence of hydrogen can act as a surfactant, enhancing adatom mobility, improving surface smoothness, and reducing the density of extended defects. As a result, the GaN layers exhibited superior crystalline integrity and improved morphological quality, underlining the potential of hydrogen-assisted MBE for fabricating high-performance GaN films.

One notable advancement was introduced by Saredin et al. [117] provided an in-depth analysis of ammonothermal GaN growth, with a particular focus on the mechanisms of defect formation and their mitigation. By systematically studying the influence of process parameters such as mineralizer concentration, temperature gradients, and pressure conditions, the authors demonstrated pathways to control both point defects and extended dislocations. Their work highlighted that careful optimization of supersaturation levels and solubility kinetics is critical for suppressing defect generation during recrystallization. Importantly, the ammonothermal crystals grown under optimized conditions exhibited full widths at half maximum of X-ray rocking curves as low as 20–30 arcsec and dislocation densities approaching 10^4^ cm^−2^, values that surpass those typically achievable in HVPE-grown GaN. These results confirm the capability of ammonothermal techniques to deliver substrates of exceptional crystalline perfection, reinforcing their role as a cornerstone for next-generation GaN optoelectronic and power devices.

Sochacki et al. [118] demonstrated a refined HVPE growth strategy that effectively limited unwanted lateral spreading of GaN during crystallization. By promoting preferential extension along the [0001] c-axis, the process yielded vertically elongated GaN structures with superior morphological stability and reduced defect formation. Crucially, this optimization maintained the inherently high deposition rates of HVPE, highlighting its potential for the scalable fabrication of thick, high-quality GaN substrates suitable for advanced device applications

A variety of epitaxial techniques have been developed for GaN growth, each characterized by distinct growth rates, defect densities, and scalability. To highlight the comparative advantages and limitations of these approaches, a summary is provided in Table 2.

As seen in Table 2, no single technique satisfies all requirements simultaneously: MOCVD remains the industrial standard, MBE provides unmatched interface control, HVPE and ammonothermal offer pathways to bulk GaN substrates, while ALD excels in conformality and precise thickness control. Ongoing research into hybrid and modified approaches (e.g., REMOCVD, MME) aims to combine these advantages and further reduce defect densities, which is critical for scaling GaN to large-area space photovoltaic applications.

## 4. Key Aspects in GaN Formation

### 4.1. Influence of Substrate Type and Quality

The substrate type and surface characteristics play a crucial role in determining the nucleation behavior, microstructural evolution, defect formation, and ultimately the functional performance of GaN layers. A notable example is provided by Santis et al. [119], who investigated the growth of cubic GaN on GaP and GaAs substrates under low-pressure MOCVD. XRD analysis revealed compressive stress in both cases, with higher structural quality achieved on GaP, as further confirmed by Raman spectra and surface morphology. The band gap of c-GaN was estimated at approximately 380 nm for both substrates, consistent with known values for the cubic phase. Extending this approach to nanoscale patterning, Li et al. [120] employed molecular dynamics simulations to evaluate GaN growth on nanopatterned AlN substrates. Introducing nanopillars significantly enhanced crystallinity by increasing the proportion of wurtzite structures and reducing dislocation density, with pillar geometry and substrate temperature identified as critical control parameters. These results demonstrate the potential of nanoscale pattern engineering to mitigate defect formation. Li et al. [121] further examined the effect of incident particle energies on crystalline quality during epitaxy. Higher energies improved surface smoothness and atomic packing but simultaneously increased dislocation nucleation height, highlighting the delicate balance between growth kinetics and internal stress. A related experimental study [122] focused on GaN growth on amorphous glass using pulsed-DC magnetron sputtering and a ZnO buffer layer. Structural analysis confirmed c-axis-oriented growth and strong near-band-edge emission, although the polycrystalline morphology and domain structure reflected inherent limitations of amorphous substrates. Dhasiyan et al. [123] employed radical-enhanced MOCVD (REMOCVD) to grow GaN on bulk GaN and GaN/Si templates. Optimizing plasma activation and minimizing radical deactivation yielded high-quality layers with XRD FWHM values as low as 72 arcsec, illustrating the strong substrate dependence of crystalline quality and the potential of low-temperature REMOCVD for GaN device structures. The influence of substrate type was also evident in the work of Kizir et al. [124], who used hollow-cathode plasma-assisted ALD to grow GaN on Si (100), Si (111), and sapphire. Although all samples exhibited wurtzite GaN, grain size, roughness, and preferred orientation varied significantly with substrate, with smoother films obtained on Si and higher growth-per-cycle values on sapphire due to enhanced nucleation behavior. Substrate surface morphology was shown to be equally critical in [125], where adjusting sapphire miscut angles enabled smooth epitaxial GaN growth under low-temperature ECR sputtering. Increasing the misorientation activated a step-flow growth mechanism, reducing surface roughness while preserving excellent crystalline quality.

Among the conventional substrates used for GaN heteroepitaxy, silicon and sapphire remain the most widely employed, but both introduce important limitations due to lattice and thermal-expansion mismatch with GaN. Sapphire offers good chemical stability and high-temperature robustness; however, the large lattice mismatch (~13–16%) and the difference in thermal-expansion coefficients generate significant tensile stress during cooldown from MOCVD growth temperatures. As the substrate and GaN layer contract at different rates, this stress promotes threading dislocations, wafer bowing, and cracking, which degrade minority-carrier lifetimes and optical performance [126]. Silicon is attractive because of its low cost, availability of large wafer diameters, and compatibility with microelectronics processing. However, the GaN/Si system suffers from an even stronger thermal-expansion mismatch. GaN expands more during growth and contracts more rapidly during cooling, leading to high tensile stress that can cause catastrophic cracking unless complex AlN/AlGaN buffer stacks are used. In addition, the large lattice mismatch (~17%) increases the density of misfit and threading dislocations, which propagate toward the surface and act as non-radiative recombination centers [127].

These mismatch-related defects have direct consequences for photovoltaic performance. They reduce carrier diffusion lengths, lower internal quantum efficiency, and accelerate degradation under thermal cycling conditions typical of space environments. Repeated temperature swings between sunlight and eclipse further increase interfacial stress, raising the risk of fatigue-related cracking and long-term device degradation. Strain accumulation also modifies polarization fields in InGaN layers, which affects carrier confinement and extraction Despite advances in GaN and InGaN growth techniques, several practical barriers remain for photovoltaic applications. A major limitation is strain accumulation caused by lattice and thermal-expansion mismatch between the epitaxial layer and the substrate. As the layer thickness increases, strain is gradually released through the formation of threading dislocations or, in extreme cases, by crack formation, especially in thick absorber layers required for efficient light absorption [128,129]. The problem becomes more pronounced for InGaN alloys with higher indium content. Increasing the In fraction lowers the bandgap and enables absorption of longer wavelengths, but it also increases lattice mismatch and internal strain. This often leads to compositional inhomogeneity, phase separation, and higher defect densities, which reduce carrier lifetime and device efficiency. As a result, there are practical limits on both the achievable indium composition and the thickness of InGaN absorber layers. Effective strain management and defect reduction therefore remain key challenges in the development of high-performance GaN-based photovoltaic devices [129,130,131].

### 4.2. GaN Doping and Challenges in p-Type Layer Formation

Doping plays a central role in tailoring the electrical properties of GaN and enabling its application in electronic and optoelectronic devices. In its undoped state, GaN is a wide-bandgap semiconductor with high resistivity, which makes the introduction of dopants essential for achieving controlled conductivity. N-type doping is comparatively well understood and widely implemented, with donors such as silicon (Si), oxygen (O), or germanium (Ge) providing high electron concentrations and stable electrical performance. These approaches have enabled the reliable fabrication of n-type GaN layers with low resistivity and excellent reproducibility. In contrast, the realization of efficient p-type doping remains highly problematic. The high ionization energy of typical acceptors (e.g., Mg), low solubility limits, and strong compensation effects from native defects or hydrogen incorporation make it difficult to achieve high hole concentrations and stable p-type conductivity. As a result, while n-type doping can be considered mature and technologically straightforward, the formation of p-type GaN layers continues to represent one of the most critical challenges in III-nitride semiconductor technology [132,133,134,135].

While Mg remains the most widely used acceptor for p-type GaN, its high activation energy, low solubility, and strong compensation by native defects severely limit hole concentrations. Recent studies have explored alternative dopants such as Zn and Be, but their incorporation often leads to poor thermal stability or self-compensation effects. To address these barriers, novel approaches are being developed, including co-doping strategies that combine different acceptors or donor–acceptor pairs to enhance activation, as well as polarization-assisted doping methods that exploit the strong internal electric fields in III-nitrides to lower ionization barriers. Modulation doping, in which carriers are supplied from adjacent layers rather than directly from the GaN matrix, has also been investigated as a way to bypass the inherent limitations of Mg. Although these techniques remain in the research stage, they represent promising directions for overcoming the long-standing challenge of achieving efficient and stable p-type conductivity in GaN [136,137,138,139,140].

These difficulties have direct implications for device performance, particularly in p–n junctions, LEDs, and other optoelectronic applications. In addition to the fundamental limitations of acceptor activation, the formation of high-quality p-type layers is further hindered by issues such as dopant activation, thermal stability, and interface degradation. Addressing these barriers requires advanced doping strategies, careful optimization of growth conditions, and effective post-deposition treatments, including thermal annealing or hydrogen passivation removal [141,142,143,144].

In photovoltaic applications, the challenges of p-type doping in GaN impact not only carrier concentration but also the ability to form low-resistance Ohmic contacts. Because of the wide bandgap and deep acceptor levels, Mg-doped GaN typically exhibits low hole mobility and limited activation, making it difficult to achieve sufficiently high conductivity in the p-layer [145]. This directly influences the contact resistance at the metal/semiconductor interface, as forming low-barrier Ohmic contacts on p-GaN is inherently challenging. As a result, the p-type contact often becomes a major contributor to the device’s series resistance. Increased series resistance reduces the Fill Factor (FF) and leads to power losses, ultimately limiting the achievable efficiency of GaN and InGaN-based solar cells. Therefore, the difficulty of creating both conductive and low-contact-resistance p-type GaN represents one of the most significant challenges for high-performance III-nitride photovoltaics [146,147].

A comprehensive analysis of these limitations was presented by Cheng [148], who focused on the activation issues associated with Mg doping with a focus on the limitations of magnesium as the primary acceptor species. Although Mg is widely used due to its compatibility with Ga in terms of electronic structure, its activation is significantly suppressed by the formation of Mg–H complexes, especially during MOCVD growth processes. As presented in the article, the presence of atomic hydrogen not only passivates Mg dopants but also leads to extremely high resistivity in the resulting layers, limiting their applicability in optoelectronic devices such as LEDs and LDs. The authors emphasized that the large covalent radius of Mg contributes to compensation effects, further reducing hole concentrations. Consequently, the formation of efficient p-type layers remains a key bottleneck, demanding the development of improved doping mechanisms and post-growth activation strategies to meet the performance requirements of advanced GaN-based devices.

Further technological challenges were discussed in [141], with particular emphasis on the fabrication of p-type regions in vertical GaN power devices, highlighting key technological and material challenges. In particular, the authors emphasized the importance of precisely controlling the effective acceptor concentration, which is the difference between the Mg dopant level and compensating donor species, including unintentional carbon (CN) and hydrogen (Hi). It was shown that hydrogen removal is critical for Mg activation, but prolonged annealing can lead to the formation of deep traps. As presented, the introduction of a p^+^ capping layer significantly enhances hydrogen out-diffusion due to the internal electric field across the p^+^/p^−^ junction, reducing required annealing time and trap formation. Furthermore, the use of selective-area Mg ion implantation and subsequent ultrahigh-pressure annealing up to 1753 K under 1 GPa nitrogen atmosphere enabled high Mg activation without GaN decomposition. The study demonstrated that Mg and H diffusion is strongly influenced by UHPA parameters and vacancy dynamics, underlining the need for precise control over annealing temperature and ambient composition.

From a structural standpoint, further insight into the nature of defects in Mg-doped GaN was offered in the work by Liliental-Weber et al. [149], who employed transmission electron microscopy to compare bulk crystals grown under high-pressure and high-temperature conditions with MOCVD-grown epitaxial layers using either continuous or δ-doping methods. The results revealed that spontaneous ordering in the form of Mg-rich planar defects appeared exclusively in N-polar bulk crystals, forming stacking-fault-like structures separated by ~10.4 nm. These defects, while resembling basal stacking faults, also exhibited features consistent with inversion domains. In contrast, growth on the Ga-polar face—characterized by a significantly faster rate—resulted in three-dimensional pyramidal and rectangular void-like defects with Mg accumulation at internal surfaces. Interestingly, similar types of polarity-dependent defects were also observed in MOCVD-grown samples with δ-doping, while continuous doping appeared to suppress their formation.

An alternative approach to achieving high-conductivity p-type GaN layers was presented in the work [150], where zinc was introduced as a transition metal dopant using the thermionic vacuum arc (TVA) technique. The resulting film exhibited a polycrystalline zincblende–wurtzite structure with promising electrical properties, including low resistivity and high hole mobility. Structural and morphological characterization confirmed uniform grain distribution (200–250 nm) and high crystalline quality. The direct bandgap was red-shifted to approximately 2.9 eV, supporting effective p-type behavior. These findings suggest that Zn-doping via TVA may serve as a viable alternative to conventional Mg-based doping for the fabrication of GaN layers in nanotechnology-driven power electronics.

### 4.3. Crystalline Defects in GaN and Strategies for Their Reduction

Defects are deviations from the ideal periodicity of a crystal lattice and are inherently present in all semiconductors. They occur in different forms: point defects (vacancies, interstitials, etc.), line defects (dislocations), planar defects (stacking faults, twins, grain boundaries), and extended defects such as cracks or voids. From a functional perspective, defects are not merely structural irregularities—they strongly influence the electrical, optical, and mechanical properties of a material. Defect-related states inside the bandgap can act as charge carrier traps or recombination centers, thereby reducing carrier lifetime, mobility, and overall device performance. In optoelectronic devices, this manifests as reduced luminescence efficiency, while in power and photovoltaic devices, it leads to higher leakage currents, increased noise, and premature degradation under stress conditions [151,152,153].

For GaN, defects represent one of the most persistent challenges despite the outstanding intrinsic properties of this wide-bandgap (3.4 eV) semiconductor. High-quality GaN substrates are still limited and expensive, which necessitates heteroepitaxial growth on foreign substrates such as sapphire, silicon carbide or silicon. The large lattice mismatch (≈16% with sapphire, ≈17% with Si, ≈3.5% with SiC) and thermal expansion coefficient differences generate high densities of threading dislocations (TDs), typically in the range of 10^8^–10^10^ cm^−2^ in heteroepitaxial GaN layers [154]. These dislocations propagate vertically through the active region and can act as non-radiative recombination centers in LEDs [155] or leakage paths in HEMTs [156]. Point defects such as nitrogen vacancies are known to create deep donor states close to the conduction band, reducing radiation hardness and long-term reliability of GaN-based devices [157].

The quality of GaN growth directly affects the performance of GaN power devices used in space energy-conversion circuits. Low dislocation density, controlled impurity incorporation, and smooth morphology are important in terms of achieving high breakdown voltages, low leakage currents as well as stable 2DEG characteristics in GaN HEMTs and Schottky diodes. Growth techniques such as MOCVD, HVPE, ELO, and pendeo-epitaxy improve uniformity and interface quality which translates into higher switching efficiency and thermal reliability. These methods support both high-quality InGaN absorbers and reliable GaN power electronics for space energy-conversion systems.

To address these issues, multiple defect-reduction strategies have been developed. A foundational approach involves the use of buffer layers, for instance, low-temperature GaN nucleation layers or AlN interlayers, which help accommodate strain and reduce dislocation density during subsequent growth. More advanced methods such as epitaxial lateral overgrowth [158,159,160] and pendeo-epitaxy redirect dislocations laterally or terminate them at mask edges, enabling a significant reduction in TD densities down to 10^6^–10^7^ cm^−2^ [161,162,163].

Epitaxial growth techniques themselves are also subject to extensive optimization for defect reduction. In metal–organic chemical vapor deposition (MOCVD), parameters such as growth temperature, V/III precursor ratio, and reactor pressure strongly influence the incorporation of point defects and the evolution of surface morphology. For example, optimized V/III ratios can suppress nitrogen vacancies and improve film stoichiometry, while higher growth temperatures generally reduce impurity incorporation, although excessively high temperatures may induce nitrogen desorption and surface roughening. Molecular beam epitaxy (MBE), although inherently slower (typically <1–2 µm/h), offers unmatched precision in controlling composition and doping profiles, which helps in engineering sharp heterointerfaces and minimizing interface-related defects. Post-growth treatments provide an additional pathway for defect management. Thermal annealing can promote defect annihilation or passivation, while chemical treatments (e.g., surface oxidation, hydrogen or plasma passivation) can neutralize electrically active defect states. Moreover, the deposition of dielectric passivation layers such as SiN_X_ or Al_2_O_3_ is often employed to suppress surface states and improve carrier lifetimes in GaN devices. Advances in defect characterization have played a crucial role in guiding these strategies. High-resolution transmission electron microscopy (HRTEM) enables direct imaging of dislocations and stacking faults, while XRD and reciprocal space mapping (RSM) provide statistical measures of dislocation densities and strain. Techniques such as cathodoluminescence (CL) and photoluminescence (PL) spectroscopy can spatially map non-radiative centers, and deep-level transient spectroscopy (DLTS) allows the identification of electrically active traps [164,165,166,167,168].

Collectively, these mitigation strategies have significantly improved GaN material quality, supporting its evolution from a laboratory curiosity to a pillar of the LED lighting revolution and a core technology in high-power RF transistors. More recently, work has begun on developing GaN components and devices with enhanced radiation tolerance, particularly for space or near-space applications. While fully mature radiation-hardened GaN photovoltaics remain at an earlier stage, the advances in defect reduction, passivation, and processing lay a solid foundation for their eventual realization. Nonetheless, defect reduction remains a central research frontier—especially for large-area, low-cost GaN substrates necessary for mass deployment in power electronics and space solar cell arrays [169,170,171].

**Figure 9:**
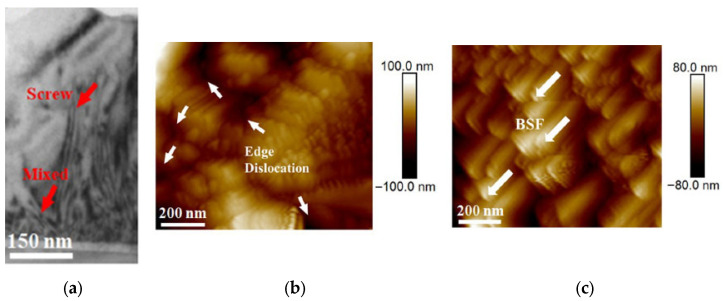
Representative experimental characterization of nonpolar GaN: (**a**) cross-sectional TEM image showing screw and mixed dislocations; (**b**) AFM image after selective etching revealing edge dislocations; (**c**) AFM image highlighting basal stacking faults (BSFs). The data demonstrate the correlation between defect density and GaN device performance. Reproduced from Ref. [173], with the permission of AIP Publishing.

Quantitative correlations between crystalline defect density and device performance have been clearly demonstrated in GaN-based heterostructures grown on different substrates. For example, Pharkphoumy et al. [172] compared AlGaN/GaN HEMTs fabricated on silicon and sapphire substrates and directly linked structural quality to electrical characteristics. High-resolution X-ray diffraction revealed significantly narrower rocking-curve full width at half maximum (FWHM) values for GaN grown on sapphire (368 arcsec) compared with silicon (703 arcsec), indicating a substantially lower threading dislocation density in the sapphire-based structures. This improvement in crystalline quality translated directly into enhanced device performance. AlGaN/GaN HEMTs on sapphire exhibited higher drain current densities (155 mA/mm vs. 150 mA/mm at V_GS_ = 0 V without passivation) and superior breakdown voltages (415 V on sapphire compared with 245 V on silicon). After SiO_2_ passivation, which suppresses surface trap-assisted leakage, the breakdown voltage increased to 425 V on sapphire and 400 V on silicon, further highlighting the combined influence of defect density and interface quality on high-field operation. The same study also demonstrated that higher defect densities induce self-heating effects and limit transconductance at elevated bias. Devices grown on silicon showed a more rapid degradation of transconductance at higher gate voltages, consistent with increased defect-assisted scattering and inferior thermal dissipation. Atomic force microscopy confirmed a much smoother GaN surface on sapphire (RMS roughness ≈ 0.88 nm) compared with silicon (≈2.96 nm), reinforcing the link between microstructural quality and carrier transport. Complementary evidence is provided by Yang et al. [173], who analyzed the influence of dislocation density on GaN-based device reliability and performance under electrically and thermally demanding conditions. Using X-ray rocking curves (XRC), cross-sectional TEM, and AFM after selective etching, the authors quantified screw and mixed dislocation densities. In Figure 9, representative XRC, cross-sectional TEM, and AFM images illustrate the direct relationship between defect type, defect density, and GaN device performance.

Clear correlations between crystalline defects and device metrics were demonstrated. Higher screw and mixed dislocation densities led to increased leakage current due to barrier-height lowering and current paths along dislocation cores, while edge dislocations and BSFs primarily reduced responsivity through enhanced carrier scattering and non-radiative recombination. Devices fabricated on GaN layers with lower overall defect densities exhibited improved electrical stability, lower dark current, and higher responsivity under high electric fields and thermal stress.

### 4.4. Substrate Reuse and Recycling Strategies for Scalable Low-Defect GaN Epitaxy

Owing to the high production cost and limited availability of freestanding GaN substrates, as well as the growing strategic importance of GaN wafer supply for next-generation electronics, several recycling and substrate-reclaim pathways have recently attracted specific attention as a strategy to reduce material scarcity risks. Although large-scale end-of-life recycling of GaN devices remains at an early stage of development, multiple substrate-reclaim approaches are relatively advanced. For instance, laser lift-off (LLO), already established in the LED industry, enables the separation of GaN epilayers from sapphire while preserving the underlying substrate for subsequent re-polishing and reuse [174]. Complementary approaches—including chemical and electrochemical lift-off, sacrificial interlayers, and selective etching processes—have been developed to detach epitaxial GaN from native GaN, SiC or sapphire while maintaining surface planarity and low defect density. Additional methods such as hydride-vapor-phase and plasma etching to remove residual GaN, together with emerging schemes aimed at regrowth on recovered templates, further demonstrate the feasibility of reducing raw-wafer consumption [175,176,177,178,179].

Beyond reclaim, several chemical recycling approaches have also been investigated to recover GaN from waste LED, RF and power-electronics devices. These methods typically involve selective leaching using acidic, alkaline, or complexing solutions that remove surface metallization and packaging residues while leaving the GaN material largely intact. Such chemical purification enables the isolation of clean GaN fragments suitable for further processing or reuse and represents a promising complementary pathway for reducing material consumption within the GaN value chain [180,181].

Importantly, these cost- and material-saving strategies interface directly with established defect-reduction techniques in GaN epitaxy—most notably epitaxial lateral overgrowth (ELO) and pendeo-epitaxy. Both methods rely on selective-area or sidewall-initiated growth to bend, block, and annihilate threading dislocations, thus lowering dislocation densities by one to two orders of magnitude compared to planar heteroepitaxy. By enabling the fabrication of low-defect-density GaN and InGaN templates on large-area, relatively low-cost substrates such as sapphire or SiC, ELO and pendeo-epitaxy significantly improve scalability for space photovoltaics [182,183,184,185,186].

When combined with substrate lift-off and reuse, these approaches offer a synergistic path toward lowering the effective cost per wafer while maintaining the high crystalline quality required for radiation-hardened, long-lifetime space-PV absorber structures. Together, defect-reduction epitaxy and substrate recycling constitute a critical technological foundation for advancing GaN-based space photovoltaics from laboratory-scale demonstrations toward economically viable multi-wafer manufacturing.

### 4.5. Semi-Polar and Non-Polar GaN Orientations

Semi-polar and non-polar GaN orientations have become increasingly important for high-In-content InGaN absorbers, as they significantly reduce or eliminate the polarization fields present in conventional c-plane (0001) GaN. Orientations such as semi-polar $\left(10\overset{-}{1}1\right)$ and $\left(11\overset{-}{2}2\right)$, as well as non-polar $\left(10\overset{-}{1}0\right)$ and $\left(11\overset{-}{2}0\right)$, suppress the quantum-confined Stark effect (QCSE), leading to improved electron–hole wavefunction overlap, enhanced radiative efficiency, and more efficient carrier extraction. This reduction in internal fields allows the incorporation of higher indium fractions without severe strain-driven defect formation, enabling InGaN bandgaps below ~1.5 eV required for the low-energy junctions in multi-junction solar cells [187,188,189,190,191].

Semi-polar GaN has already demonstrated improved crystalline quality in high-In InGaN layers compared with c-plane growth, including narrower photoluminescence linewidths, reduced compositional non-uniformity, and lower defect densities. Recent studies also show that semi-polar orientations can sustain thicker InGaN layers without phase separation and exhibit higher critical thicknesses before relaxation occurs, further underscoring their relevance for high-performance InGaN photovoltaic absorbers [192,193,194].

### 4.6. Growth-Related Limitations and Their Impact on GaN Photovoltaic Device Architectures

The feasibility of advanced GaN-based photovoltaic concepts, such as tandem architectures and InGaN multi-quantum-well (MQW) active layers, is strongly constrained by material and growth limitations. Precise control of indium composition is required to achieve the desired bandgap sequence in tandem cells; however, increasing indium content leads to higher strain, compositional fluctuations, and defect formation, which limit both layer thickness and crystalline quality [195,196].

Similarly, high defect densities reduce carrier lifetimes and diffusion lengths, directly affecting current collection in thick InGaN layers and MQW solar cells. Challenges in achieving highly conductive p-type GaN with low contact resistance further increase series resistance, which is particularly detrimental for multi-junction devices requiring efficient current matching. As a result, growth-related constraints play a decisive role in determining which GaN-based device architectures are practically achievable for space photovoltaics.

Beyond structural and compositional constraints, growth-related defects also strongly influence the electrical transport and dynamic behavior of GaN-based devices. Quantitative data reported for commercial and near-commercial GaN devices clearly demonstrate that electrical transport and dynamic behavior are strongly governed by material quality, defect density, and heterointerface engineering. GaN-based devices typically exhibit critical electric fields in the range of ~3.0–3.5 MV cm^−1^; however, studies consistently show that increased threading dislocation density (TDD ≥ 10^8^–10^9^ cm^−2^) results in leakage currents that are 2–3 orders of magnitude higher and in premature electrical breakdown compared with low-defect GaN structures (TDD ~ 10^6^ cm^−2^) [197,198]. Dynamic effects are similarly sensitive to defects and interface traps. Reported increases in dynamic on-resistance remain below ~10–15% for low-defect GaN devices, but can reach 30–100% in structures with high trap densities, particularly under high electric fields and elevated operating temperatures [199,200]. These effects are strongly linked to charge trapping at GaN/AlGaN heterointerfaces and at p-GaN/metal contacts, which directly affect switching behavior and energy losses. Junction temperature further modulates carrier transport and device stability. Experimental data show that GaN devices can maintain stable operation up to ~150–200 °C; however, higher temperatures significantly exacerbate trapping, current collapse, and long-term degradation mechanisms, especially in defect-rich structures [201,202]. Recent reports also indicate that optimized heterostructure design—such as controlled AlGaN thickness (≈8–15 nm), Al composition (x ≈ 0.2–0.3), and Mg-doped p-GaN layers (~10^19^ cm^−3^)—is essential to balance electric field distribution, leakage suppression, and dynamic stability [203,204]. Collectively, these studies demonstrate that breakdown strength, defect density, heterointerface quality, and thermal stability jointly define both the current limitations and the performance potential of GaN-based devices. These parameters are therefore critical for the design of reliable, low-loss power-conditioning and energy-harvesting systems intended for space photovoltaic applications.

## 5. GaN-Based Solar Cell Architectures for Space Applications

The unique physical properties of GaN and its ternary alloys, particularly InGaN, have stimulated intensive research into advanced solar cell architectures for space environments. Among these, InGaN/GaN heterojunctions are especially promising because the bandgap of InGaN can be tuned across a wide spectral range (0.7–3.4 eV) simply by varying the indium concentration. This bandgap flexibility makes it theoretically possible to absorb nearly the entire solar spectrum within a single material system. Laboratory studies of InGaN/GaN single-junction devices have reported respectable conversion efficiencies under AM0 conditions, while also highlighting their superior resistance to displacement damage by protons and electrons. Compared to GaAs and InP cells, which degrade significantly under comparable fluences, InGaN/GaN devices retain a higher fraction of their initial performance, suggesting a distinct advantage for long-duration missions in harsh radiation environments [203,204,205].

To place GaN-based photovoltaic devices in the broader context of space-relevant energy harvesting technologies, a comparative overview of representative electrical performance metrics is provided in Table 3. The table summarizes key parameters reported for different energy harvesting approaches, including maximum current and voltage, power density, charge density, and typical operating frequencies. Such a comparison highlights the distinct operational regimes and application niches of photovoltaic, piezoelectric, triboelectric, and thermoelectric systems.

The data indicate that different energy harvesting technologies operate in fundamentally distinct regimes, with piezoelectric and triboelectric devices favoring high-voltage or dynamic excitation scenarios, while photovoltaic systems provide continuous power under steady illumination. GaN-based photovoltaics combine the intrinsic advantages of photovoltaic operation with high radiation tolerance and thermal stability, making them particularly attractive for space environments. This positions GaN photovoltaics as a complementary solution to other harvesting approaches rather than a direct replacement.

Quantum well (QW) structures further extend the design potential of GaN-based photovoltaics. By embedding thin layers of InGaN within wider-bandgap GaN barriers, multi-quantum-well (MQW) devices can confine carriers and enhance the probability of radiative recombination while allowing precise tailoring of absorption and emission wavelengths. These structures have been explored both for improved current generation in solar cells and for hybrid optoelectronic applications such as luminescent down-shifting layers. Importantly, MQW solar cells have demonstrated strong tolerance to ionizing radiation, showing minimal losses in photoluminescence and carrier mobility even after proton fluences exceeding 10^14^ cm^−2^. This resilience highlights the potential of QW engineering not only for enhancing efficiency but also for ensuring long-term stability in orbit [212,213,214,215,216].

The development of multijunction GaN-based solar cells represents another major avenue of research. Multijunction devices stack layers of varying composition to capture different portions of the solar spectrum, thereby surpassing the Shockley–Queisser limit of single-junction cells. Wide-bandgap GaN or InGaN top cells can serve as radiation-hard, high-energy absorbers, while narrower-bandgap InGaN layers or hybrid III–V junctions can capture longer-wavelength photons. Recent modeling studies suggest that InGaN-based tandem cells could, in principle, achieve efficiencies exceeding 40% under AM0 illumination, rivaling state-of-the-art III–V triple-junction devices but with potentially higher radiation hardness and thermal stability. Although experimental demonstrations remain limited by growth challenges, progress in epitaxy and compositional control continues to close this gap [203,217,218].

**Figure 10:**
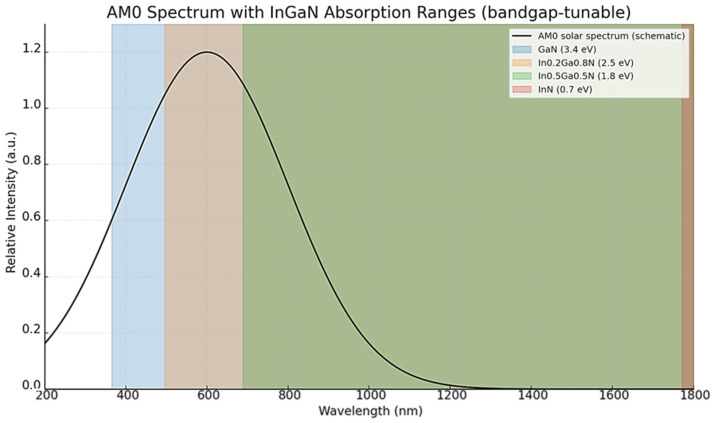
Schematic illustration of the AM0 solar spectrum (black curve) compared with the absorption ranges of InGaN alloys at different indium contents.

The tunability of the InGaN bandgap from 0.7 eV to 3.4 eV enables absorption across nearly the entire solar spectrum under AM0 conditions. This remarkable flexibility makes InGaN/GaN alloys ideally suited for designing multi-junction architectures, where current matching and spectral utilization are essential for maximizing efficiency. Figure 10 schematically illustrates the AM0 solar spectrum together with the absorption ranges corresponding to different InGaN compositions.

As shown in Figure 6, the compositional tuning of InGaN enables nearly complete spectral coverage, a feature that is unmatched among conventional III–V semiconductors. This property represents a cornerstone of GaN-based tandem design strategies. By tuning the indium fraction, the bandgap can be engineered from 3.4 eV (GaN) to 0.7 eV (InN), thereby enabling nearly full coverage of the solar spectrum. This bandgap flexibility underpins the design of InGaN/GaN multi-junction and tandem solar cells for space applications.

Bandgap engineering through compositional grading provides another critical design strategy. By gradually varying the indium composition across InGaN/GaN interfaces, strain relaxation can be controlled, which reduces threading dislocation densities and improves crystal quality. Such graded layers also enable smoother energy band transitions, which facilitate carrier transport and improve current matching in tandem architectures. In addition, compositional grading has been shown to enhance absorption in targeted spectral ranges, helping to optimize device performance without sacrificing structural integrity. These methods, combined with advanced epitaxial growth approaches such as plasma-assisted MBE and metal–organic CVD, are essential for scaling GaN-based solar cells to wafer-level production suitable for space applications [219,220,221].

Overall, while GaN-based photovoltaics are less mature than the commercial III–V multijunction cells currently deployed on satellites, they provide a unique combination of tunable bandgap, exceptional radiation hardness, and high thermal stability. These features make them highly attractive candidates for next-generation solar power systems in low Earth orbit, geostationary orbit, and deep-space missions. Continued advances in heterostructure engineering, defect reduction, and large-area substrate development will be decisive in transitioning GaN-based photovoltaic devices from laboratory prototypes to operational space-qualified solar arrays.

Recent studies show that GaN and other wide-bandgap materials such as AlGaN/GaN, AlScN/GaN, and Ga_2_O_3_ are becoming important also for advanced optoelectronic and neuromorphic devices. For example, AlGaN/GaN synaptic transistors can be tuned by light and are able to reproduce key biological synaptic behaviors, enabling neuromorphic vision processing with high accuracy [222]. Ga_2_O_3_-based solar-blind ultraviolet photodetectors can also function as artificial synapses, offering tunable conductance, very low energy consumption, and strong recognition capability for neuromorphic computing tasks [223]. Further developments include solar-blind photodetectors based on SrTiO_3_/AlGaN heterostructures, which demonstrate low dark current and high responsivity while accurately performing neural-network recognition tasks [224]. In addition, CuO_x_/AlGaN nanowire photoelectrochemical devices can achieve external quantum efficiencies above 100% through multiple exciton generation, making them promising for self-powered UV imaging and weak-light detection in space applications [225].

Bias-reconfigurable AlScN/GaN photodetectors can switch between fast UV detection, synaptic behavior, and encrypted optical imaging within a single device, showing how wide-bandgap heterostructures can support multifunctional, intelligent hardware for future aerospace and autonomous systems [226].

Despite highly optimistic theoretical predictions indicating that InGaN-based tandem solar cells could achieve efficiencies exceeding 40%, experimental realizations remain at an early stage of development. To date, laboratory-scale InGaN/GaN solar cells have demonstrated maximum power conversion efficiencies in the range of approximately 3–5%, with the highest reported values obtained for optimized multiple-quantum-well (MQW) architectures with relatively low indium content. Most experimentally reported devices exhibit efficiencies between 1% and 3%, limited primarily by insufficient absorption in thin InGaN layers, high defect densities, and polarization-induced carrier separation. Recent experimental studies employing advanced device designs, including MQW absorbers, polarization engineering, and improved carrier extraction schemes, have reported efficiencies approaching ~4–5% under laboratory conditions; however, these values remain far below both theoretical limits and the performance of established space-qualified III–V photovoltaic technologies [227,228,229,230,231].

Experimentally reported InGaN/GaN solar cells exhibit a relatively wide spread of photovoltaic parameters, reflecting strong sensitivity to indium composition, layer thickness, defect density, and device architecture. In laboratory-scale single-junction and MQW-based InGaN/GaN devices, typical open-circuit voltages (Voc) range from approximately 1.5 to 2.6 V, benefiting from the wide bandgap of III-nitrides. Short-circuit current densities (Jsc) are generally limited to ~1–5 mA·cm^−2^, primarily due to incomplete absorption in thin InGaN layers and polarization-induced carrier separation. Fill factors (FF) reported for experimentally realized devices typically fall in the range of 40–65%, with reductions attributed to series resistance, limited p-type conductivity, and non-radiative recombination at defects and interfaces [232,233,234]. In contrast to laboratory-scale InGaN/GaN photovoltaic devices, GaInP/GaAs-based multi-junction solar cells represent a technologically mature and space-qualified solution. Experimental studies report power conversion efficiencies exceeding 30%, with demonstrated values reaching ~31–32% for GaInP/GaAs/Ge triple-junction architectures under space-relevant or near-space illumination conditions. The reported current–voltage characteristics correspond to cumulative open-circuit voltages on the order of ~2.5–2.7 V, short-circuit current densities in the range of ~14–17 mA·cm^−2^, and fill factors typically exceeding ~75%, reflecting optimized current matching and low resistive losses in mature III–V multi-junction architectures. These experimentally demonstrated photovoltaic parameters underline a substantial performance gap between current InGaN/GaN solar cells and incumbent GaInP/GaAs-based technologies employed in space power systems [235,236].

The experimentally observed limitations in open-circuit voltage, short-circuit current density, and fill factor of InGaN/GaN solar cells are primarily attributed to two fundamental material-related bottlenecks. The first is phase separation and compositional inhomogeneity in high-indium-content InGaN layers, which leads to indium clustering, localized bandgap fluctuations, and enhanced non-radiative recombination, ultimately reducing carrier collection efficiency and fill factor [237,238]. The second major limitation arises from strong spontaneous and piezoelectric polarization fields inherent to the wurtzite III-nitride system. These internal electric fields induce spatial separation of electrons and holes along the growth direction, reducing wavefunction overlap and carrier extraction efficiency, particularly in quantum-well-based absorber structures [239,240,241]. Together, phase separation and polarization-induced field effects constitute the dominant obstacles preventing InGaN/GaN solar cells from simultaneously achieving high Voc, high Jsc, and high fill factor, thereby explaining the persistent gap between experimental performance and theoretical efficiency limits [242,243].

For clarity, a quantitative comparison of experimentally reported photovoltaic parameters for InGaN/GaN solar cells and incumbent space-qualified GaInP/GaAs-based technologies is summarized in Table 4.

As summarized in Table 4, experimentally demonstrated InGaN/GaN solar cells currently operate far below their theoretical efficiency limits, with substantially reduced short-circuit current densities and fill factors compared to incumbent GaInP/GaAs-based multi-junction technologies. This quantitative comparison highlights the pronounced performance gap between emerging nitride-based photovoltaic concepts and mature space-qualified III–V solar cells. Consequently, further progress in InGaN/GaN photovoltaics critically depends on overcoming fundamental material limitations related to indium incorporation, defect density, and polarization effects.

## 6. GaN Wafers in Space Applications: Market Context, Radiation Environment, and System-Level Implications

### 6.1. GaN Wafers in Space

The GaN wafers market by itself has been recording a strong momentum in the recent years, caused by increasing demand in such industries as automotive (inc. EV), telecommunications (5G, 6G), LED (microLED) and optoelectronics. According to the Future Market Report, the GaN wafers market value was estimated at USD 850 million in 2024 and is believed to reach USD 2250 million by 2032 (CAGR 13.4%). Similarly, the GaN wafer foundry market covering semiconductors and related services is expected to surge from USD 1.78 billion in 2024 to USD 3.12 billion by 2032 (CAGR 8.2%). As far as market segments are concerned, a freestanding GaN (GaN-on-GaN) wafer market—a premium one due to its lowest defect densities and higher performance (RF, advanced optoelectronics, vertical GaN devices)—is forecasted to grow from USD 1.0~1.5 billion in 2024 to USD 3.0~3.5 by 2032/2033. Despite the fact that freestanding GaN wafers are expected to reflect a minor fraction of the total global wafer production, they are believed to deliver disproportionately higher strategic and technological value in performance-sensitive device architectures.

GaN wafers are becoming increasingly important in the space sector, especially for space applications that require outstanding performance under extreme conditions, as this technology combines high energy efficiency, resistance to harsh conditions and the potential for system miniaturization. Its material properties such as

- high breakdown voltage,
- thermal stability, and
- electron mobility

make GaN a perfect material for space applications where extreme environment conditions impose rigorous performance demands.

GaN devices enable next-generation space missions by delivering superior power efficiency, radiation hardness and operational reliability that exceed the capabilities of conventional semiconductor materials like Si. GaN with its core properties seems to be exceptionally well-suited for the hard conditions encountered in space environments. Boasting a wide bandgap of approximately 3.4 eV, GaN devices remain reliable at high temperatures and sustain high power densities without electrical breakdown. This capability is crucial for space systems, which must constantly perform in extreme thermal fluctuations, i.e., from the deep cold of outer space to the intense heat induced by direct solar irradiation.

As mentioned above, GaN in contrast to silicon-based devices can operate at high temperatures thanks to its wide bandgap. Its thermal robustness can be enhanced by GaN’s high electron mobility, enabling rapid electron transport within the crystal lattice. Therefore GaN devices in space may secure superior high-speed and high-frequency performance, making them ideal for advanced communication and radar systems essential to modern space industry [244].

What is more, GaN’s high breakdown voltage provides a huge advantage in space-grade power electronics. Components based on GaN can sustain substantially higher voltages than silicon devices, making them highly suitable for applications demanding precise voltage regulation and enhanced power efficiency. During space missions, where power consumption is critically constrained and battery longevity is crucial, this efficiency edge is critical for ensuring mission reliability and success.

There is one factor characteristic for GaN that might be the most important for space applications—exceptional tolerance to ionizing radiation. The space environment is saturated by high-energy particles originating from cosmic rays and solar events, which can cause degradation or catastrophic failure in conventional semiconductor devices. GaN’s intrinsic radiation hardness affords substantial mitigation against these effects, markedly lowering the likelihood of radiation-induced malfunction. This resistance makes GaN a highly suitable material for long-duration and deep-space missions, where sustained electronic reliability is a top priority [245].

GaN technology distinguishes itself from other materials such as Si through a vast range of advantages and tangible benefits in spacecraft design and operation. High efficiency is the most important advantage, as GaN-based power amplifiers and transistors can achieve efficiency levels that minimize power consumption while maximizing battery life. In energy-constrained space environments, where solar panels and batteries are significant factors in weight and cost, this efficiency improvement could enable longer missions.

The compact and lightweight design of GaN devices resolves key problems and challenges in space systems, where every kg sent into orbit increases launch costs. Because GaN components are much lighter and smaller than silicon-based ones, spacecraft engineers can reduce the overall mass without making compromises, and they can even enhance system performance. GaN capability for high-frequency operation is especially valuable for communication systems, radar and sensing instruments used in space missions. With satellite communications shifting to higher-frequency bands to achieve greater bandwidth, GaN’s efficient performance at these elevated frequencies becomes increasingly necessary. The integration of GaN technology into space systems may face some technical challenges, such as thermal management, which seems to be critical due to GaN’s capability for high power density. Despite the fact that GaN devices may tolerate higher temperatures, effective thermal design is essential to maximize device performance and lifespan. This is especially challenging in space, where (within a vacuum) some innovative approaches need to be implemented in order to dissipate heat.

What really needs to be emphasized is the fact that due to GaN’s high switching frequency capability, it is possible to reduce the passive component sizes of power management architectures, and it may bring consequent savings in their mass and volume. That is why system engineers must resolve electronic interference (EMI) issues to stay compatible and functionally integrated with other onboard systems. Moreover, application of GaN material and devices into spacecraft missions and satellites may cause significant and complex challenges and problems, because, despite having superior electrical characteristics, GaN components require perfect integration with existing spacecraft architectures and ground support infrastructure. In order to achieve smooth operation and integration, a specialized knowledge in high-frequency circuit design is necessary, and advanced thermal management strategies and comprehensive system-level optimization are required [246].

### 6.2. The Space Radiation Environment

The space radiation environment is known for setting hard challenges for all materials; however, GaN technology overcomes them better and more effectively than conventional materials used in semiconductors. Long exposure to cosmic rays, solar particle events and trapped radiation within planetary magnetospheres causes various degradation mechanisms in electronic components, including:

(a) Single-event effects (SEE);
(b) Total ionizing dose (TID) damage;
(c) Displacement damage.

GaN power device technology enables a new generation of power converters for operation in harsh radiation conditions of space. GaN devices operate at higher frequencies, higher efficiencies, and greater power densities than ever achievable before. These power devices can exhibit superior radiation tolerance compared with silicon MOSFETs depending upon specific device design [34].

Moreover, GaN wide bandgap and resilient crystal lattice secure internal resistance to these radiation effects, and thanks to its wide bandgap reduces susceptibility to ionizing radiation, while the material’s robust atomic structure mitigates displacement damage caused by high-energy particles. This inherent radiation resistance decreases reliance on heavy shielding, enabling weight reduction and streamlined spacecraft architecture [247].

The global radiation-hardened electronics market size accounted for USD 1.85 billion in 2024 and is predicted to increase from USD 1.96 billion in 2025 to approximately USD 3.24 billion by 2034, expanding at a CAGR of 5.76% from 2025 to 2034. The market extends to chip designers, fabrication facilities, and radiation testing labs. Radiation-hardened electronics include electronic components and systems designed or processed to tolerate ionizing radiation and operate reliably in high-radiation environments using hardened process technologies, design mitigation, shielding, and fault-tolerant architectures. Growth in the radiation-hardened electronics market is driven by the inexorable need to safeguard critical systems against the pernicious effects of cosmic rays, solar flares, and nuclear radiation [248].

Radiation-hardened electronics constitute components and systems engineered to withstand:

(a) Ionizing radiation through hardened semiconductor processes;
(b) Radiation-tolerant circuit design techniques;
(c) Shielding strategies;
(d) Fault-tolerant system architectures.

The market growth is enhanced by high industry pressure and demand for solutions protecting important hardware from cosmic radiation, solar flares and nuclear radiation. Space exploration and military upgrades are speeding up, and therefore, the demand for the mentioned technologies surges. Radiation-hardened systems have evolved from specialized resilience solutions into essential enablers of national security capabilities and scientific missions. Their applications include orbital platforms and terrestrial command-and-control systems, where operational failure is unacceptable. Accordingly, the market is underlined by the core requirements of durability, functional integrity and defense-grade reliability.

GaN has become the fastest-growing material in the industry, providing greater performance and reliability for power electronics applications. Owing to its wide bandgap property, GaN can realize high-voltage, high-temperature and high-frequency operation with low performance degradation. GaN devices possess intrinsic radiation hardness, placing the technology as one of the top candidates for upcoming aerospace and defense applications. Key applications are satellite power-conditioning units, advanced radar modules and electric propulsion subsystems demonstrating its functional benefits as well as the disruptive nature of the technology [249].

However, radiation effects are not entirely eliminated in GaN devices and still require precise device design and selection tailored to the mission-specific radiation environment. Choosing GaN components with validated radiation tolerance is critical for ensuring mission reliability. Continued research aims to enhance GaN radiation hardness further and produce specialized radiation-hardened variants for the most extreme space environments.

## 7. Conclusions and Perspectives

GaN has established itself as a highly promising material platform for space photovoltaics due to its wide bandgap, strong radiation hardness, and superior thermal and chemical stability. These intrinsic properties address the key limitations of conventional III–V compounds such as GaAs and InP, which exhibit significant degradation under proton and electron irradiation and limited robustness during thermal cycling. Through advances in epitaxial growth techniques including MOCVD, MBE, HVPE, and ALD, substantial progress has been achieved in reducing defect densities, controlling composition, and engineering sharp heterointerfaces. The tunability of InGaN alloys across nearly the full solar spectrum further enhances the versatility of GaN-based devices, opening pathways toward high-efficiency heterostructures tailored for space environments.

Nevertheless, several challenges remain unresolved. Persistent difficulties in realizing efficient and stable p-type doping, the high threading dislocation densities associated with heteroepitaxial growth, and the scalability of cost-effective large-area substrates continue to limit practical deployment. Multijunction GaN-based solar cells and InGaN/GaN quantum well structures have been shown to offer theoretical efficiencies above 40% under AM0 illumination, yet experimental demonstrations are still constrained by epitaxial and compositional complexities.

Looking ahead, the integration of defect-reduction methods, compositional grading strategies, and novel passivation schemes is expected to play a decisive role in bridging the gap between laboratory prototypes and flight-ready modules. With sustained progress in materials science, device engineering, and space-relevant testing, GaN is positioned to evolve from a complementary technology into a cornerstone of future space photovoltaic power systems.

In addition, recent market analyses indicate that the technological momentum of GaN extends far beyond photovoltaics, with rapidly expanding opportunities driven by the growth of electric vehicles, renewable energy systems, 5G communications, IoT electronics, and aerospace applications. Continued advances in wafer scaling, cost reduction, and GaN–Si integration are expected to further broaden the application landscape, reinforcing the long-term strategic importance of GaN-based materials across both terrestrial and space technologies.

## Figures and Tables

**Table 1:** Comparison of selected material properties of GaN, GaAs, and InP relevant to space photovoltaic applications. Values are given for room temperature (300 K) unless otherwise noted. Radiation tolerance values are indicative and depend on particle energy, device architecture, and test conditions.

Property	GaN	GaAs	InP
Bandgap Eg (eV)	≈3.4 (direct)	≈1.42 (direct)	≈1.34 (direct)
Thermal conductivity κ (W·m^−1^·K^−1^)	130–230 (quality-dependent; up to ~300 reported) [60]	~55–56	~68
Radiation tolerance (illustrative)	InGaN/GaN MQW: minimal degradation after proton fluences ≥10^14^ cm^−2^ [60]	Pronounced performance loss under proton/electron irradiation [10]	Significant degradation even at fluences ~10^13^ cm^−2^ (energy-dependent) [61]
Typical degradation under protons/electrons	Slower efficiency loss due to high displacement threshold energy; MQW more robust [60]	Faster efficiency loss; high density of radiation-induced defects [10]	Better than GaAs in some regimes, but still notable degradation [61]
Availability/toxicity	Contains Ga and N; nitrogen is non-toxic. Gallium is listed as a critical raw material (CRM) in EU assessments [62]	Arsenic-bearing (toxicity, handling and EHS burdens) + CRM considerations for Ga/As supply [62]	Indium-bearing (critical raw material; supply concentration) + P toxicity handling [62]

**Table 2:** Comparison of epitaxial growth techniques for GaN, including typical growth rates, dislocation densities, scalability, and common substrates.

Growth Method	Typical Growth Rate *	Dislocation Density *	Scalability	Typical Substrates	Remarks
MOCVD	1–5 µm/h	~10^8^–10^9^ cm^−2^ (on sapphire/Si)	High—industry standard	Sapphire, SiC, Si, GaN templates	Mature technology, excellent uniformity, but costly precursors and high T (~1000 °C)
MBE	≤2 µm/h	~10^8^ cm^−2^ (can be lower with optimized buffers)	Limited to small wafers (≤6″)	Sapphire, SiC, GaN	Excellent interface control, sharp heterostructures, slow and expensive
HVPE	50–200 µm/h	10^5^–10^7^ cm^−2^ (depending on buffer/substrate)	Medium—thick GaN and bulk growth	Sapphire, SiC, GaN, freestanding GaN	Very high growth rate, used for bulk GaN
Ammonothermal	~20–200 µm/day	10^4^–10^5^ cm^−2^ (lowest reported)	Low (lab scale, bulk crystals)	Native GaN seeds	Produces high-quality GaN, very low TDs, but slow and costly
ALD (including PEALD, HCPA-ALD)	0.05–0.2 nm/cycle	Template-dependent	Low–medium (research, conformal films)	Sapphire, Si, GaN templates	Precise thickness/composition control, low T (≤400 °C), very slow growth
Other variants (LP-MOCVD, LA-MOCVD, REMOCVD, MME)	Comparable to MOCVD (optimized for specific goals)	Typically 1 order of magnitude lower than conventional MOCVD	Research scale	Sapphire, SiC, GaN	Emerging modifications improving TD reduction, interface quality, or growth uniformity

* Values depend strongly on reactor design, process parameters, and particularly on the choice of substrate and the architecture of buffer layers (e.g., low-temperature nucleation layers, AlN interlayers, or superlattices). These factors critically influence strain relaxation and the propagation of dislocations in GaN films.

**Table 3:** Comparison of representative electrical performance parameters reported for selected energy harvesting technologies, including piezoelectric (PENG), thermoelectric (TEG), triboelectric (TENG), conventional photovoltaic (PV), and GaN-based photovoltaic devices.

Type of Device	Maximum Current mA	Maximum Voltage, V	Power Density **	Operating Frequencies
Piezoelectric (PENG) [206]	0.24	128.0	7390 W/m^3^	Several Hz to tens of kHz
Thermoelectric (TEG) [207,208]	18.50	11.3	86,400 W/m^3^	-
Triboelectric (TENG) [209]	0.67	1221.0	0.066 W/cm^2^	Typically 1 to tens of Hz
Photovoltaic (PV) [210]	18.20 *	3.1	0.040 W/cm^2^	-
GaN-based photovoltaic [211]	22.20 *	1.0	0.003 W/cm^2^	-

* Current density values reported per unit area (mA·cm^−2^). ** Power density is reported per unit volume (W·m^−3^) for bulk energy harvesters (PENG, TEG) and per unit area (W·m^−2^) for surface-based devices (TENG, PV).

**Table 4:** Experimentally reported photovoltaic parameters of InGaN/GaN solar cells compared with incumbent space-qualified III–V multi-junction technologies.

Material System	Device Type	Efficiency, %	V_OC_, V	J_SC_, mA · cm^−2^	FF, %
InGaN/GaN	Single-junction	1–3	1.5–2.6	1–5	40–65
InGaN/GaN	MQW	up to ~5	2.0–2.6	2–5	45–65
GaInP/GaAs/Ge	Triple junction	~31–32	~2.5–2.7	~14–17	>75

## Data Availability

No new data were created or analyzed in this study.

## Abbreviations

The following abbreviations are used in this manuscript:

AFM	Atomic Force Microscopy
ALD	Atomic Layer Deposition
AO	Atomic oxygen
AR	Augmented Reality
BSF	Basal stacking fault
CAGR	Compound Annual Growth Rate
CRM	Critical raw material
CL	Cathodoluminescence
CTE	Coefficient of thermal expansion
DC-DC	Direct Current to Direct Current
DLTS	Deep-level transient spectroscopy
ECR	Electron cyclotron resonance
EHS	Environment, Health and Safety
ELO	Epitaxial lateral overgrowth
ESS	Energy storage system
EV	Electric vehicle
EW	Electronic warfare
FF	Fill Factor
FWHM	Full width at half maximum
GEO	Geostationary Earth orbit
GSMA	Global System for Mobile Communications
GPC	Growth per cycle
HEMTs	High-electron-mobility transistors
HCPA-ALD	Hollow-cathode plasma-assisted atomic layer deposition
HRTEM	High-resolution transmission electron microscopy
HVPE	Hydride vapor phase epitaxy
IEA	International Energy Agency
IEL	Ionizing energy loss
LA-MOCVD	Laser-assisted MOCVD
LD	Laser Diode
LED	Light-Emitting Diode
LLO	Laser lift-off
LP-MOCVD	Low-pressure MOCVD
MBE	Molecular beam epitaxy
MEO	Medium Earth orbit
MIMO	Multiple-Input Multiple-Output
MME	Metal-modulated epitaxy
MOSFET(s)	Metal–oxide–semiconductor field-effect transistor(s)
MOCVD	Metal–organic chemical vapor deposition
MQW	Multiple quantum well
NIEL	Non-ionizing energy loss
OBC	On-board charger
PD	Power Delivery
PEALD	Plasma-enhanced ALD
PENG	Piezoelectric Nanogenerator
PL	Photoluminescence
PSU	Power Supply Unit
QCSE	Quantum-confined Stark effect
REMOCVD	Radical-enhanced metalorganic chemical vapor deposition
RF	Radio-Frequency
RHEED	Reflection High-Energy Electron Diffraction
RSM	Reciprocal space mapping
SAED	Selected area electron diffraction
TD	Threading dislocation
TEG	Thermoelectric Generator
TEM	Transmission electron microscopy
TENG	Triboelectric Nanogenerator
TWh	Terawatt-hours
TVA	Thermionic vacuum arc technique
UHV	Ultra-high vacuum
USB-C	Universal Serial Bus Type-C
UV	Ultraviolet
VR	Virtual Reality
XPS	X-ray Photoelectron Spectroscopy
XRC	X-ray rocking curves
XRD	X-ray diffraction
XRR	X-ray Reflectivity
